# Diversity and abundance of filamentous and non-filamentous “*Leptothrix”* in global wastewater treatment plants

**DOI:** 10.1128/aem.01485-24

**Published:** 2025-02-14

**Authors:** Karina Seguel Suazo, Marta Nierychlo, Zivile Kondrotaite, Francesca Petriglieri, Miriam Peces, Caitlin Singleton, Jan Dries, Per H. Nielsen

**Affiliations:** 1 Biochemical Wastewater Valorization and Engineering (BioWAVE), Faculty of Applied Engineering, University of Antwerp 26660 , Antwerp, Belgium; 2 Center for Microbial Communities, Department of Chemistry and Bioscience, Aalborg University 489197 , Aalborg, Denmark; Colorado School of Mines, Golden, Colorado, USA

**Keywords:** activated sludge, filamentous bacteria, *Leptothrix*, *Rubrivivax*, *Ideonella*, FISH

## Abstract

Species belonging to the genus *Leptothrix* are widely distributed in the environment and in activated sludge (AS) wastewater treatment plants (WWTPs). They are commonly found in iron-rich environments and reported to cause filamentous bulking in WWTPs. In this study, the diversity, distribution, and metabolic potential of the most prevalent *Leptothrix* spp. found in AS worldwide were studied. Our 16S rRNA amplicon survey showed that *Leptothrix* belongs to the general core community of AS worldwide, comprising 32 species with four species being most commonly found. Their taxonomic classification was re-evaluated based on both 16S rRNA gene and genome-based phylogenetic analysis showing that three of the most abundant “*Leptothrix”* species represented species in three other genera, *Rubrivivax*, *Ideonella*, and the novel genus, *Ca*. Intricatilinea. New fluorescence *in situ* hybridization (FISH) probes revealed rod-shaped morphology for the novel *Ca*. Rubrivivax defluviihabitans and *Ca*. Ideonella esbjergensis*,* while filamentous morphology was found only for *Ca*. Intricatilinea gracilis. Analysis of high-quality metagenome-assembled genomes revealed metabolic potential for aerobic growth, fermentation, storage of intracellular polymers, partial denitrification, photosynthesis, and iron reduction. FISH in combination with Raman microspectroscopy confirmed the *in situ* presence of chlorophyll and carotenoids in *Ca*. Rubrivivax defluviihabitans and *Ca*. Intricatilinea gracilis. This study resolves the taxonomy of abundant but poorly classified “*Leptothrix”* species, providing important insights into their diversity, morphology, and function in global AS wastewater treatment systems.

**IMPORTANCE:**

The genus *Leptothrix* has been extensively studied and described since the 1880s, with six species currently described but with the majority uncultured and undescribed. Some species are assumed to have a filamentous morphology and can cause settling problems in wastewater treatment plants (WWTPs). Here, we revised the classification of the most abundant *Leptothrix* spp. present in WWTPs across the world, showing that most belong to other genera, such as *Rubrivivax* and *Ideonella*. Furthermore, most do not have a filamentous morphology and are not problematic in WWTPs as previously believed. Metabolic reconstruction, including some traits validated *in situ* by the application of new fluorescence *in situ* hybridization probes and Raman microspectroscopy, provided additional insights into their metabolism. The study has contributed to a better understanding of the diversity, morphology, and function of “*Leptothrix*,” which belong to the abundant core community across global activated sludge WWTPs.

## INTRODUCTION

The bacterial genus *Leptothrix* from the family *Comamonadaceae* occurs in different natural environments, including water iron seeps, iron mat biofilm, and soil samples (1). In such habitats, its presence is commonly associated with iron and manganese oxidation (2). Currently, six species have been described based on phenotypic characteristics, that is, *L. cholodnii*, *L. discophora*, *L. mobilis*, *L. ochracea*, *L. pseudo-ochracea*, and *L. lopholea*. However, only four reference strains belonging to *L. cholodnii*, *L. discophora*, and *L. mobilis* are available for comparative studies from public culture collections (3). Furthermore, *Leptothrix* spp. have also been observed in activated sludge (AS) wastewater treatment plants (WWTP) (4). The identification of *Leptothrix* spp. has traditionally been based on morphological features and ecological aspects, such as filamentous morphology and formation of sheath encrusted with iron, which is often referred to as the *Sphaerotilus–Leptothrix* group of sheathed bacteria among AS studies (4, 5).

The role of *Leptothrix* in AS systems has received considerable attention since members are reported to cause filamentous bulking sludge (6–8) in municipal and industrial WWTPs, strongly impairing effluent quality (7, 9, 10). They may also be the predominant cause of filamentous bulking during the early stage of granulation in plants with aerobic granules (11). Furthermore, members of *Leptothrix* are common and abundant across the world, as the genus belongs to the general core community of full-scale activated sludge WWTPs (12). Studies where *Leptothrix* were identified by morphology (4) indicate that they are commonly involved in bulking, whereas recent studies with DNA-based identification have not confirmed this in municipal plants in Denmark (13) and industrial plants in Belgium (14). These contradictions suggest that there is no clear evidence of a negative influence on sludge settleability.

Isolated strains of *L. cholodnii* are chemolithoautotrophs capable of oxidizing reduced iron compounds (Fe^2+^), while species of *L. discophora* are iron- and manganese-oxidizing bacteria. The morphological features of *Leptothrix* from culture collection strains (3) have been described as straight-rod cells with dimensions of 0.6 to 1.5 µm width by 1.5 to 14 µm length, with a characteristic rough sheath surface. Cells can appear as single, pairs, or chains of cells forming filaments. Limitation of carbon (C), nitrogen (N), phosphorus (P), or vitamins in pure culture media may result in filamentous morphology (15).

In the past, *Leptothrix* and *Sphaerotilus* were classified as one group (16–18), which frequently led to misidentification. *Leptothrix* was later taxonomically separated (5) and has been extensively studied in natural environments (1, 19, 20). Discrepancies in the taxonomic classification of “*Leptothrix*” spp. caused by the close phylogenetic relationship of the genus *Leptothrix* with *Sphaerotilus*, *Rubrivivax*, and other members of the *Comamonadacea* family have been increasingly highlighted in the literature (21–23). Siering et al. (24) suggested the need to revise the taxonomy and nomenclature of *L. discophora*, as some strains of this species, based on the 16S rRNA gene, clustered closer to the genus *Rubrivivax* than to other close cultivated relatives of *Leptothrix*. In a recent study, the taxonomy of the family *Comamondaceae* was revised using genome-based phylogenetic analysis, resulting in the reclassification of *L. mobilis* as a novel member of the genus *Sphaerotilus* (25).

The aim of this study was to investigate the diversity, abundance, and metabolic potential of the most prevalent members of the 16S rRNA-defined genus “*Leptothrix*” in AS systems worldwide. We re-evaluated the taxonomic classification of the “*Leptothrix*” spp. based on both 16S rRNA gene and genome-based phylogenetic analysis, resulting in the proposal for one new genus and two new species . The potential filamentous morphology of several abundant species was studied using novel fluorescence *in situ* hybridization (FISH) probes. Insights into their metabolic potential were obtained using high-quality metagenome assembled genomes (HQ MAGs) (26), and FISH–Raman microspectroscopy was used to investigate different metabolic traits *in situ*.

## RESULTS AND DISCUSSION

### Comparative genomics redefines the classification of the most abundant “*Leptothrix*” spp. in WWTPs

**Fig 1:**
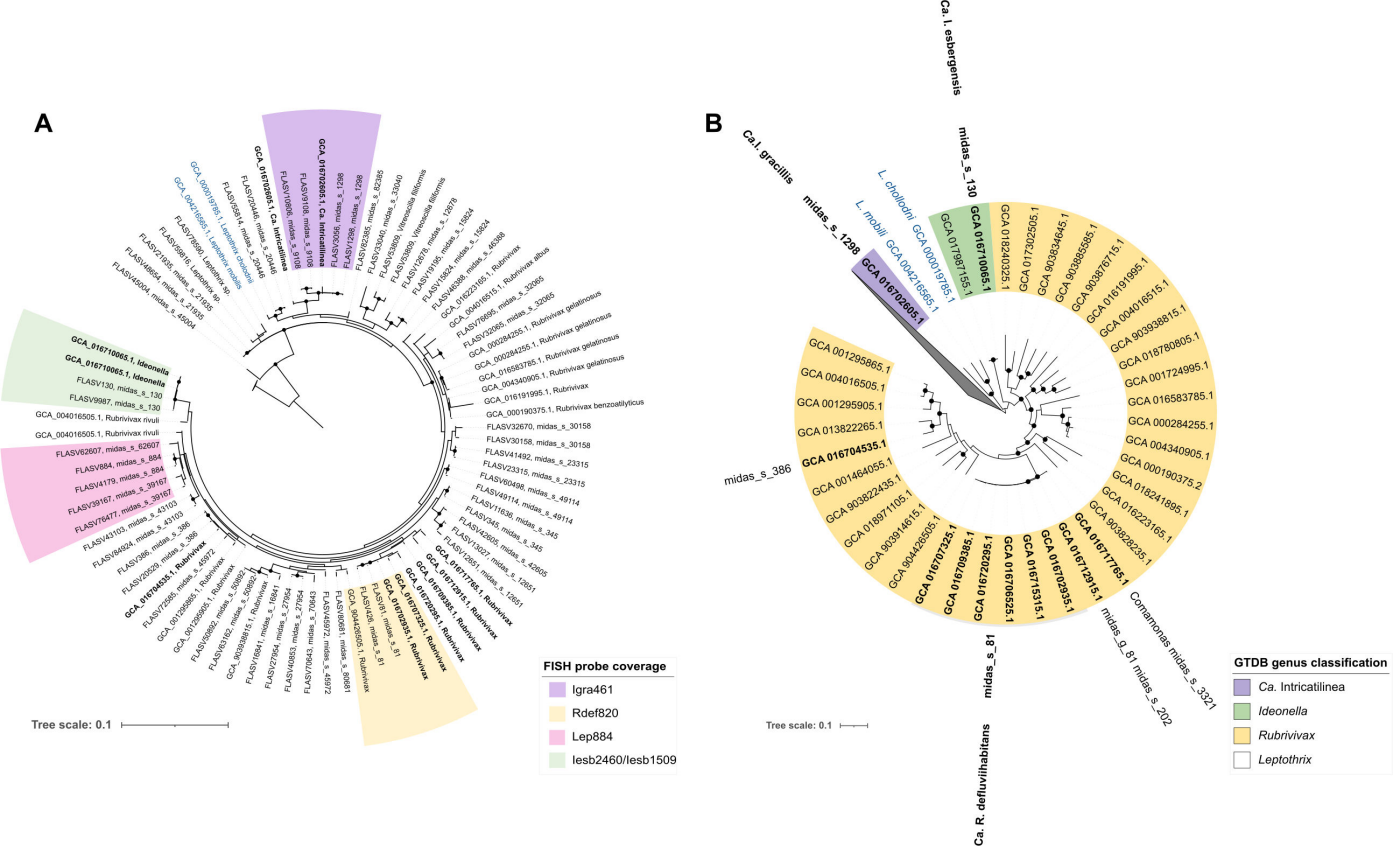
Phylogenetic and phylogenomic trees of “*Leptothrix*” spp. (**A**) Maximum-likelihood 16S rRNA gene tree showing diversity of all species classified as “*Leptothrix”* by the MiDAS4.8 database (FLASV) and extracted from the related HQ MAGs (GCA). 16S rRNA genes from HQ MAGs from Danish AS WWTPs (26) are shown in bold. GTDB taxonomy is shown where applicable for 16s rRNA genes from HQ MAGs. Coverage of the new FISH probes is indicated with color boxes. Bootstrap support >70% from 1,000 re-sampling is indicated by the black circles. Sequences from genus *Lautropia* belonging to the same order (*Burkholderiales*) were used as the outgroup. (**B**) Maximum-likelihood genome tree (bootstrap support >95% is indicated by the black circles) created from the concatenated alignment of 120 single-copy marker gene proteins extracted from HQ MAGs from Danish WWTPs (in bold) and GTDB database. The color boxes indicate different genera based on the GTDB-Tk classification. In both trees, *Leptothrix* isolates are shown in blue, and the scale bar represents substitutions per nucleotide base.

Phylogenetic analysis of the most abundant “*Leptothrix*” spp. in global WWTPs (see below) was performed using full-length 16S rRNA gene sequences from the ecosystem-specific MiDAS4 reference database (12) combined with 16S rRNA gene sequences classified as *Ideonella*, *Rubrivivax*, and *Leptothrix* extracted from the set of 1,083 HQ MAGs recovered from Danish WWTPs (26) and from the Genome Taxonomy Database (GTDB)-Tk database (27). This analysis revealed that “*Leptothrix*” spp. clustered into three distinct genera (Fig. 1A).

Phylogenomic analysis (Fig. 1B) using the HQ MAGs from Danish WWTPs (26) and genomes from the GTDB was used to determine the genome-based taxonomy of *Leptothrix* midas_s_81 (6 MAGs), midas_s_130 (1 MAG), midas_s_1298 (1 MAG), and midas_s_386 (1 MAG), whereas no MAGs were found to represent the abundant species midas_s_884. For a better representation of the “*Leptothrix*” group, additional 29 MAGs from GTDB representing the genera *Rubrivivax*, *Ideonella*, and *Leptothrix* were included in the genome tree together with two MAGs recovered from Danish WWTPs (26). These two clustered with other midas_s_81 MAGs by GTDB taxonomy, despite 16S rRNA gene-based classification identifying them as *Comamonas* and a novel genus midas_g_81.

None of the 16S rRNA gene-defined “*Leptothrix*” species clustered with the genomes of *Leptothrix* isolates and, thus, should not be classified as *Leptothrix*. All *Leptothrix* midas_s_81 MAGs clustered within the genus *Rubrivivax* representing one novel species. The MAG representing midas_s_130 grouped with the genus *Ideonella* also representing a novel species, while the MAG representing midas_s_1298 corresponded to a novel genus within the family *Burkholderiaceae*. All the species studied clustered within the family *Burkholderiaceae* (order *Burkholderiales*, class Gammaproteobacteria), while the taxonomy based on 16S rRNA genes located these genera within the family *Comamondaceae* (order *Burkholderiales*). The genera closest to the family *Comamonadaceae* are known to be taxonomically uncertain and have been referred to as genera *incertae sedis* (28, 29), highlighting the need to fully resolve these lineages.

To determine the taxonomic classification, the full-genome average nucleotide identity (ANI) analysis was performed with proposed boundaries for genus (75–77%) and species (>95%) levels (30, 31). However, neither ANIb nor ANIm showed clear division between the different genera proposed by GTDB-Tk (Fig. S1). Due to the morphological and physiological differences (discussed later) between the abundant “*Leptothrix*” spp. analyzed, we decided to reclassify them based on the GTDB-Tk taxonomic classification. Based on their characterization presented in this study, for the two new species and one new genus, we propose new candidate names. For midas_s_81, we propose the name *Ca*. Rubrivivax defluviihabitans*,* for midas_s_130, *Ca*. Ideonella esbjergensis, and for midas_s_1298, which represents a novel genus, we propose the name *Ca*. Intricatilinea gracilis. We refer to the reclassified group of species as “*Leptothrix.*” Details of the taxonomic proposals can be found in Tables S1 to S3.

Investigation of metabolic potential, morphology, and *in situ* presence of storage polymers (shown later) revealed substantial differences across the genera studied supporting the proposed taxonomic classification into the family *Burkholderiaceae* (see below).

### Global distribution and abundance of “*Leptothrix”* in full-scale AS plants

The distribution and abundance of “*Leptothrix*” spp., including the newly reclassified species found in AS systems, were investigated using the amplicon data from the recent MiDAS4 global survey (12). The observed “*Leptothrix”* abundance was highly dependent on primer choice, with V4 underestimating their abundance compared to V1–V3 primers (Fig. S2). This was particularly pronounced for the most abundant species, midas_s_884 and *Ca*. Id. esbjergensis (midas_s_130), which were not detected with the V4 primer set. Therefore, we used the V1–V3 amplicon data set to further study their distribution and abundance across 929 AS samples from 30 countries.

**Fig 2:**
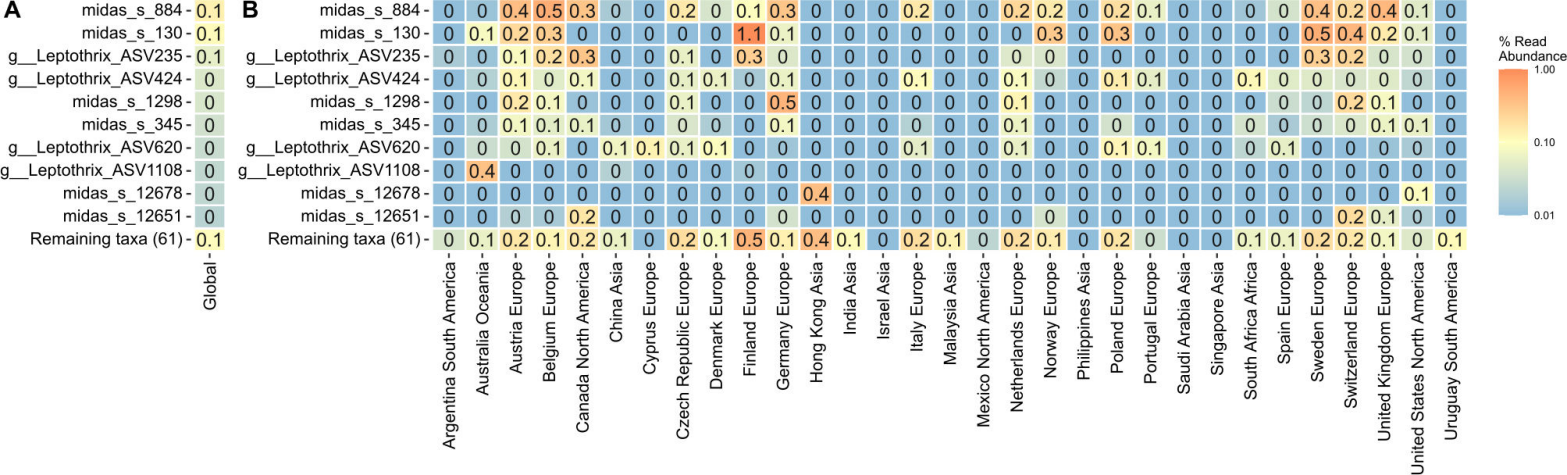
Relative abundance of top 10 “*Leptothrix*” spp. in full-scale WWTPs. (**A**) Mean relative abundance in global full-scale WWTPs. (**B**) Mean relative abundance in full-scale WWTPs across different countries (ordered by continent). Analyzed data originated from MiDAS4 survey (12).

The 16S rRNA gene-defined genus “*Leptothrix”* was part of the general core community (12), ranking ninth out of the 51 core genera, comprising 32 species, with four considered abundant. None of the “*Leptothrix*” spp. found in AS corresponded to previously described isolates, such as *L. ochracea* or *L. mobilis*. Only undescribed and uncultured species were found in global WWTPs, all with MiDAS placeholder names. The abundant species included: *Leptothrix* midas_s_884, followed by *Ca*. Id. esbjergensis (midas_s_130) and *Ca*. In. gracilis (midas_s_1298) (Fig. 2). In addition, *Ca*. R. defluviihabitans (midas_s_81) was among the abundant “*Leptothrix”* spp*.* present in Danish AS plants (Fig. S3) and also included in the analysis. Additionally, 41 unclassified ASVs were observed, stressing that a number of species were poorly resolved by the 16S rRNA gene. Interestingly, they were most abundant in Europe and hardly present in Asia and South America.

**Fig 3:**
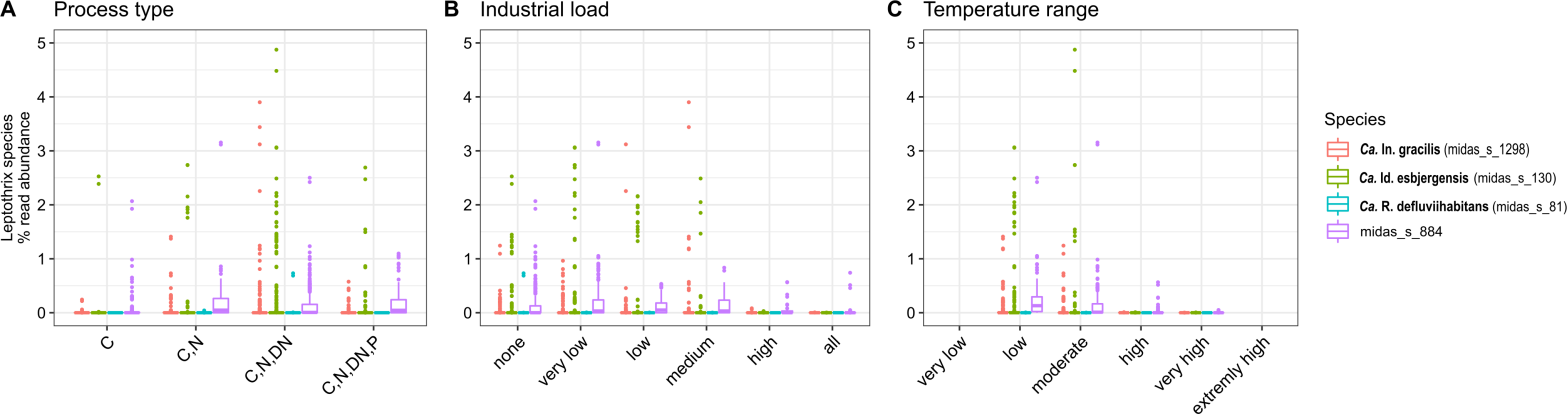
Abundance distribution of “*Leptothrix*” spp. across global WWTPs based on: (**A**) process type (C—carbon removal, C,N—carbon removal with nitrification, C,N,DN—carbon removal with nitrification and denitrification, C,N,DN,P—carbon, nitrogen, and biological phosphorus removal), (**B**) industrial load (none = 0%, very low = 0–10%, low = 10–30%, medium = 30–50%, high 50–100%, all = 100%), and (**C**) temperature range (very low ≤10°C, low = 10–15°C, moderate = 15–20°C, high = 20–25°C, very high = 25–30°C, extremely high ≥30°C); exact Kruskall–Wallis values are presented in Table S4.

We evaluated the correlation between the abundance of the dominant species and the four main process types, temperature in the WWTPs, and industrial load (Fig. 3). The non-parametric Kruskal–Wallis test showed significant differences among process types (*P*-value < 0.05, details in Table S4) for all the analyzed species (Fig. 3). Higher abundance was found in more advanced plants, such as those with biological nitrogen removal (Fig. 3A), suggesting their potential role in nutrient transformation as further confirmed by metabolic potential (see below). Industrial load also caused significant differences in the abundance of *Ca*. Id. esbjergensis, *Ca*. In. gracilis, and midas_s_884 with highest abundances observed in plants with none to medium industrial load (<50%), while no significant effect was observed for *Ca*. R. defluviihabitans (Fig. 3B). Furthermore, temperature was observed to influence the abundance of all species, except *Ca*. R. defluviihabitans*,* with the highest abundances encountered in low and moderate temperature ranges (Fig. 3C).

### Seasonal dynamics of “*Leptothrix*” spp. in four Danish WWTPs

**Fig 4:**
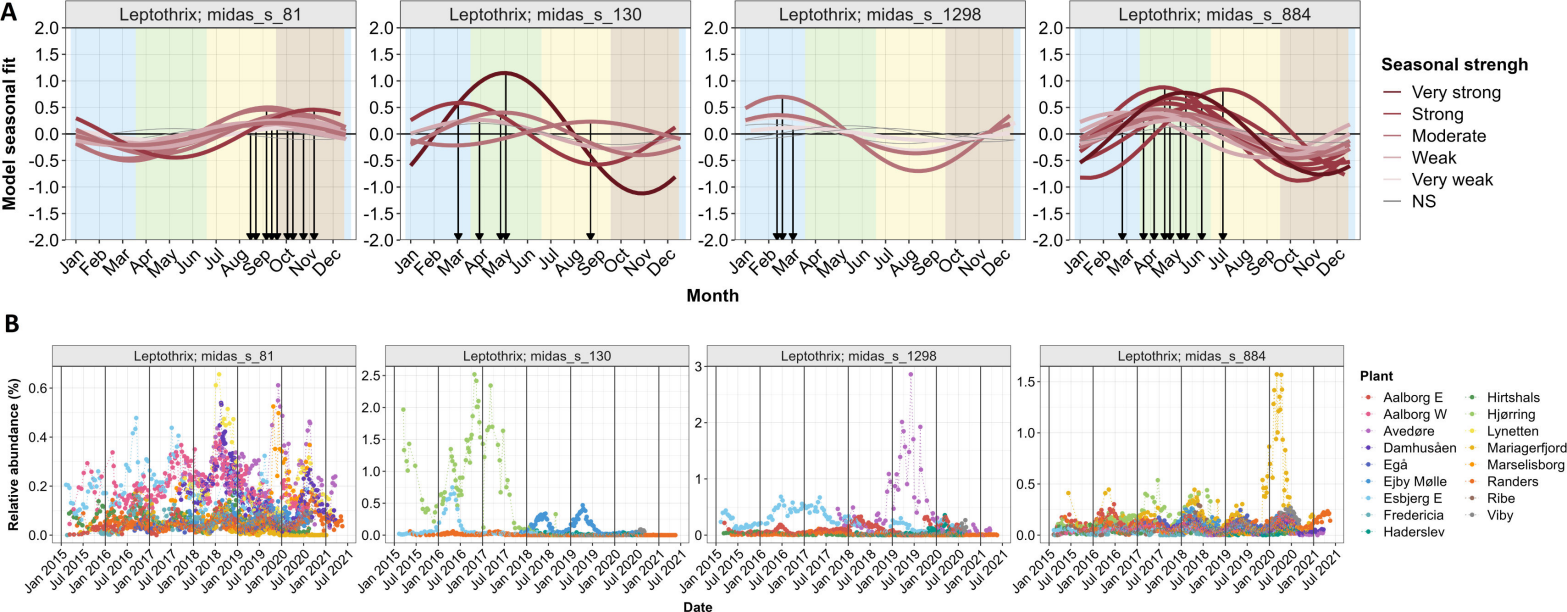
Seasonal dynamics of “*Leptothrix*” spp. (**A**) Time-series abundance in four Danish WWTPs. (**B**) Fitted seasonal component: color intensity of the fitted trend represents the seasonal component’s strength, while the arrows and numbers (week) indicate peak abundance. Each line represents a WWTP with Aalborg E = AAE, Aalborg W = AAW, Damhusåen = DAM, and Randers = RND; NS—not significant.

Since many bacterial taxa in Danish AS plants are showing yearly seasonal variation (32), we analyzed the seasonal dynamics of four abundant “*Leptothrix*” spp. using 6 years of long time-series data from four Danish WWTPs. The raw time-series (Fig. 4A) were decomposed in three main components (trend, season, and residuals). The seasonal component was further fitted to a harmonic model (Fig. 4B) to evaluate the strength of the seasonality and to estimate the peak abundance (32). *Leptothrix* midas_s_884 and *Ca*. R. defluviihabitans showed moderate to strong recurrent seasonal patterns across all WWTPs analyzed, with midas_s_884 showing peak abundance between April and June (spring season) (Fig. 4A) and *Ca*. R. defluviihabitans between August and November (autumn season) (Fig. 4B). Both seem prevalent at low and moderate temperatures, representative of the spring and autumn seasons. *Rubrivivax* has previously been detected at low temperatures (33). The remaining species, *Ca*. Id. esbjergensis and *Ca*. In. gracilis, were only found in one or two plants. *Ca*. Id. esbjergensis showed a rather strong seasonality, whereas seasonal variations for *Ca*. In. gracilis were very weak or not significant (Fig. 4B), though the results could not be confirmed in other WWTPs.

### Visualization and *in situ* characterization

New 16S rRNA FISH probes were designed using the MiDAS4 database, targeting *Ca*. R. defluviihabitans, *Ca*. In. gracilis, and *Leptothrix* midas_s_884. For *Ca*. Id. esbjergensis, species-specific probes were designed targeting the 23S rRNA gene. Each probe targeted only one morphology, supporting that the probes are species-specific.

**Fig 5:**
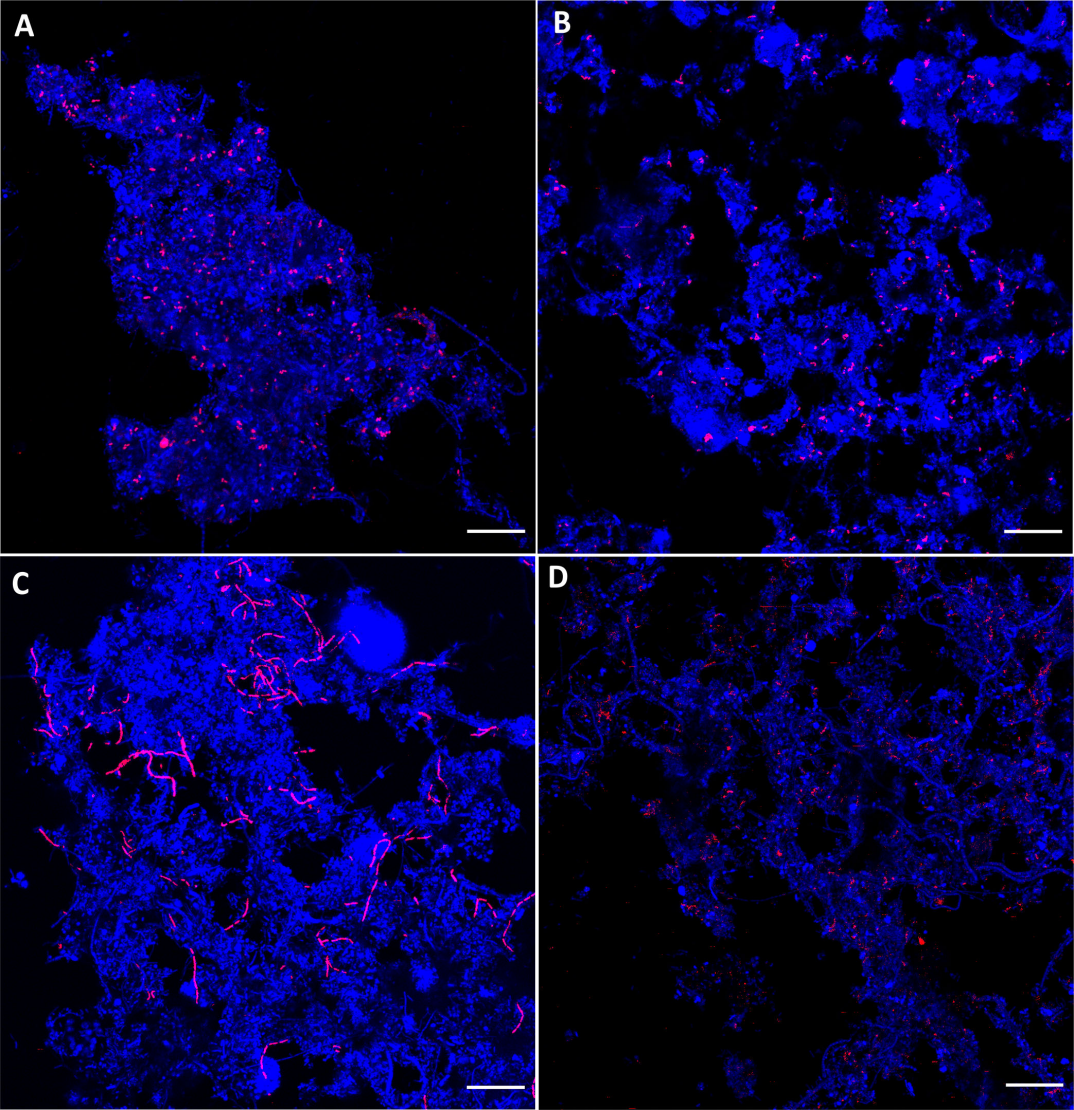
Composite FISH micrographs of “*Leptothrix*”. (**A**) *Leptothrix* midas_s_884 hybridizing with probe Lep884, (**B**) filamentous *Ca*. Intricatilinea gracilis hybridizing with probe Igra461, (**C**) *Ca*. Rubrivivax defluviihabitans hybridizing with probe Rdef820, and (**D**) *Ca*. Ideonella esbjergensis hybridizing with probe Iesb1509. Specific probe is shown in magenta; other bacteria hybridizing with probe EUBmix are shown in blue. Scale bar is 20 µm.

All targeted species showed a different cell morphology than the sheathed filament *Leptothrix* is known for. *Leptothrix* midas_s_884 formed rod cells (0.4–0.7 µm × 0.9–1.9 µm) located within the activated sludge flocs (Fig. 5A). Only *Ca*. In. gracilis cells had filamentous morphology with relatively short and very thin filaments (0.4–0.6 µm × 15–57 µm) (Fig. 5B), clearly shorter than the known sheathed filaments of *Leptothrix*. Their location within the flocs suggests a positive structural role rather than contributing to bulking episodes. *Ca*. R. defluviihabitans formed rod-shaped cells (0.3–0.6 µm × 0.7–1.4 µm) radially symmetric with rounded ends observed mostly scattered within the flocs and, in some cases, clustering in microcolonies (Fig. 5C). *Ca*. Id. esbjergensis formed straight and long rod-shaped cells (0.2–0.6 µm × 1.3–3.2 µm) mostly scattered within the sludge flocs (Fig. 5D). Cell shape and dimensions of *Ca*. R. defluviihabitans and *Ca*. Id. esbjergensis are similar to other *Rubrivivax* (34) and *Ideonella* (35) species.

The biovolume fraction from the quantitative FISH (qFISH) analysis was compared with the relative read abundance of amplicon sequencing for all four species (Table 1). *Leptothrix* midas_s_884 had similar biovolume and read abundance fractions. *Ca*. In. gracilis and *Ca*. R. defluviihabitans had higher biovolume fraction than relative read abundance, whereas *Ca*. Id. esbjergensis had higher relative read abundance than fraction observed in the biovolume. This may reflect the difference in copy number of the 16S rRNA gene in the MAGs, one and two, respectively (Table S5).

**TABLE 1:** Abundance estimation by 16S rRNA amplicon and qFISH for each species (average percentage)

Species new/old name	FISH probe	Sample WWTP, date	Abundance %	
			Amplicon sequencing	qFISH
–/*Leptothrix* midas_s_884	Lep884	Viborg, 2011-02-04	1.3	2.4 ± 1
		Odense, NE 2016-03-01	1.1	1.1 ± 0.4
		Skive, 2006-05-06	0.7	<0.5
*Ca*. Intricatilinea gracilis/*Leptothrix* midas_s_1298	Igra461	Esbjerg W 2014-02-04	0.9	<0.5
		Aalborg E 2015-02-10	0.5	2.5 ± 0.9
		Esbjerg E 2011-05-06	0.5	1.2 ± 0.4
*Ca*. Rubrivivax defluviihabitans/*Leptothrix* midas_s_81	Rdef820	Esbjerg W, 2006-08-19	0.6	2.1 ± 0.8
		Damhusåen, 2018-11-15	0.5	0.9 ± 0.4
		Lynetten, 2018-11-13	0.3	1.2 ± 0.4
*Ca*. Ideonella esbjergensis/*Leptothrix* midas_s_130	Iesb2460	Esbjerg, W 2015-10-18	3.8	0.5 ± 0.2
		Hjørring, 2016-10-30	2.4	<0.5
		Odense NE, 2018-02-28	0.6	<0.5
*Ca*. Ideonella esbjergensis/*Leptothrix* midas_s_130	Iesb1509	Esbjerg W, 2015-10-28	3.8	<0.5
		Hjørring, 2016-10-30	2.4	1.1 ± 0.3
		Odense NE, 2018-02-28	0.6	<0.5

For midas_s_884, we were unable to validate its taxonomy based on GTDB classification as no HQ MAG is available for this species. The FISH probe Lep884 bound to rod-shaped single cells. The filamentous morphology of *Leptothrix* spp. has been well described in environmental samples, but this has been poorly confirmed in engineered systems, such as AS WWTPs. *L. discophora* can be visualized by the FISH probe LDI (8), which hybridizes with two morphotypes, including rod-shaped single cells. The identification of *L. discophora* in industrial AS WWTPs using the LDI probe has been reported (9) but without clear evidence of their filamentous morphology. *In silico* assessment of the LDI probe using the MiDAS4 database revealed that it targets several genera in the family *Comamonadaceae* (with more than 50% of target sequences belonging to genus *Hydrogenophaga*), including three sequences from the genus *Leptothrix*. Two of these belong to *Ca*. Id. esbjergensis (rod-shaped), while the last belongs to midas_s_33040, which was not observed in high abundance in any of the data sets available. This suggests that the filamentous bacteria targeted by the LDI probe belong to one of the other *Comamonadaceae* genera. To date, despite several studies indicating that “*Leptothrix”* filaments may cause bulking in AS WWTPs (8, 9), none of these studies provide reliable identification or evidence of their filamentous morphology or proposed negative effect on sludge settling.

**Fig 6:**
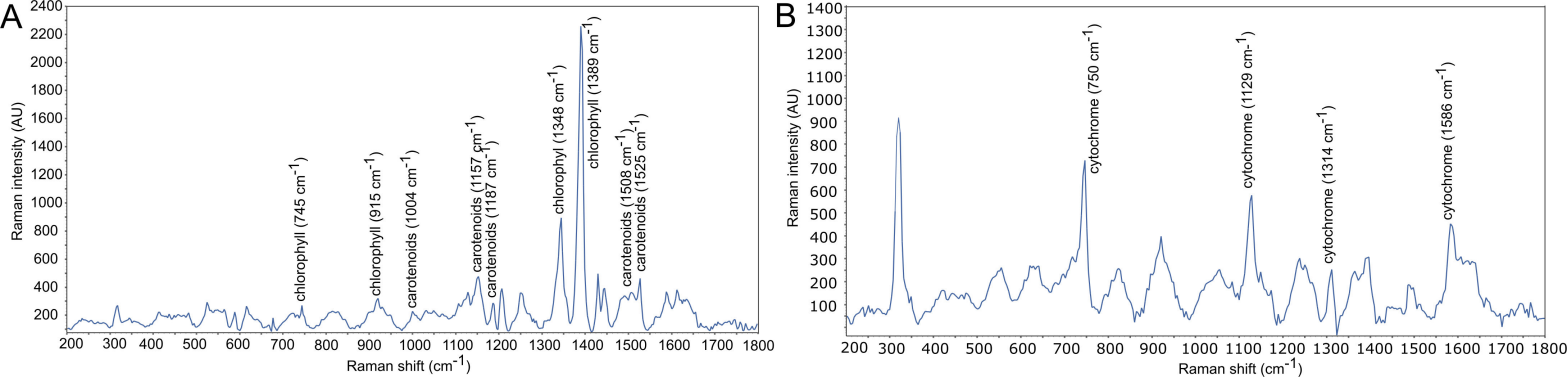
Examples of Raman spectra from FISH-resolved bacterial cells. (**A**) *Ca*. Rubrivivax defluviihabitans, which is also representative for midas_s_884 and *Ca*. Intricatilinea gracilis, and (**B**) *Ca*. Ideonella esbjergensis.

We applied the new FISH probes in combination with Raman microspectroscopy to investigate the presence of polyphosphate (poly-P) and other storage polymers (26, 36, 37), see Fig. 6. Interestingly, the Raman spectra of *Ca*. R. defluviihabitans, *Ca*. In. gracilis, and *Leptothrix* midas_s_884 showed several peaks attributable to the presence of chlorophyll (745, 915, 1,348, and 1,389 cm^−1^) and carotenoids (1,008, 1,157, 1,187, 1,508, and 1,524 cm^−1^) in the cells (Fig. 6A). Similar spectra have previously been observed in other *Rubrivivax* species, as well as other photosynthetic microorganisms (38–43). Spectra of FISH-defined cells belonging to *Ca*. Id. esbjergensis were instead characterized by cytochrome c peaks (750, 1,129, 1,314, and 1,586 cm^−1^), which generally indicates activity linked to electron transport, and these peaks have been previously detected in bacteria involved in denitrification or metal reduction processes (44, 45) (Fig. 6B).

### Metabolic potential of novel species and genus

**Fig 7:**
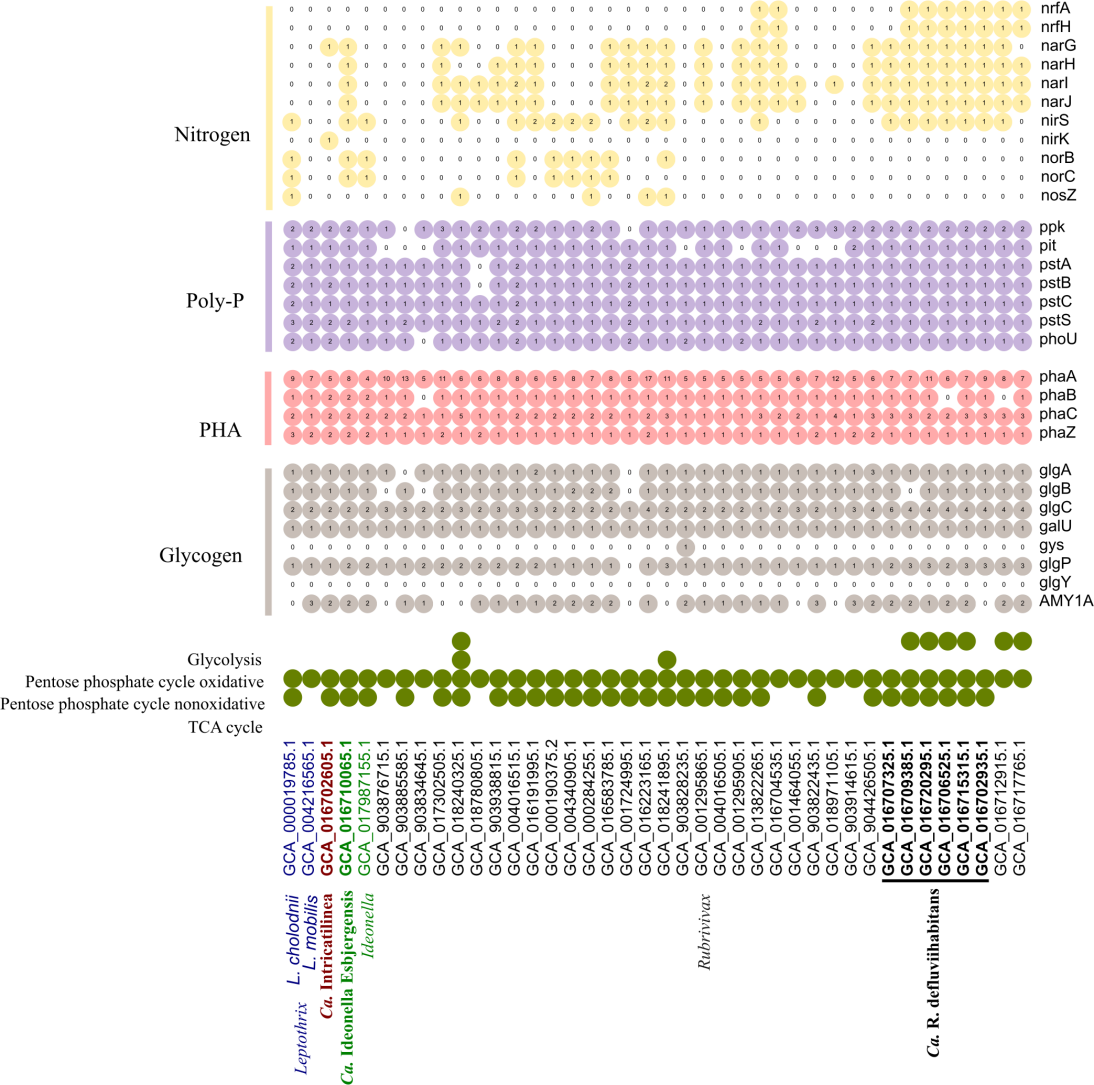
Metabolic potential of *Leptothrix*, *Ca*. Intricatilinea, *Ideonella*, and *Rubrivivax* MAGs, with a focus on nutrient removal physiologies. The species of interest (*Ca*. In. gracilis, *Ca*. Id. esbjergensis, and *Ca*. R. defluviihabitans) are marked in bold. The numbers in colored circles represent gene copy numbers. For the full list of gene names and associated KO numbers, see the supplemental material (Table S5).

The novel species belong to the genera *Ideonella*, *Rubrivivax*, and the novel genus *Ca*. Intricatilinea, for which no information related to their metabolism in AS systems exists. We investigated all MAGs for specific metabolic traits potentially important in AS plants, including carbon degradation, accumulation of storage polymers, and denitrification (Fig. 7). We also aimed to determine how the novel species differ in metabolic potential by including distinctive metabolism, such as photosynthesis-related genes known for *Rubrivivax* spp. (34), or iron cycling genes described for *Leptothrix* spp. (46).

The glycolysis pathway, Embden–Meyerhof–Parnas (EMP) [Kyoto Encyclopedia of Genes and Genomes (KEGG) module: M00001], was predicted only in MAGs from *Ca*. R. defluviihabitans. The non-oxidative pentose phosphate pathway (M00007) was predicted in all species analyzed, while the full pentose phosphate pathway (M00004) was not predicted in any of the MAGs. Complete tricarboxylic acid cycle (M00009) was found in *Ca*. In. gracilis, *Ca*. Id. esbjergensis, and *Ca*. R. defluviihabitans. *Ca*. Id. esbjergensis and *Ca*. In. gracilis had a potential for sugar uptake as they encoded transporter genes for glucose/mannose (*gts*) and glycerol 3-phosphate (*ugp* and *malK*). Additionally, *Ca*. In. gracilis had fructose (*frc*) transporter genes. All MAGs contained the fructose degradation gene (*scrK*), with *Ca*. Id. esbjergensis also encoding genes for sucrose degradation (*malZ*). All species had the potential for amino acid uptake, as evidenced by the high copy numbers of genes for branched amino acids (*liv*) transport. Additionally, genes for the transport of peptides (*dpp*) and glutamate/aspartate (*glt*) were encoded by *Ca*. Id. esbjergensis and *Ca*. In general, L-amino acid (*aap*) genes were found in *Ca*. In. gracilis and *Ca*. R. defluviihabitans. The MAGs of all species also encoded genes for the degradation of amino acids, including arginase (*arg*), aromatic amino acids (*paaABCDE* and *paaK*), and L-serine (*sdaA*). Tryptophan (*tnaA*) degradation was also encoded by *Ca*. R. defluviihabitans. This suggests that amino acids may serve as an important energy source.

All MAGs encoded the acetate transporter gene (*actP*) and the *acs* gene, indicating the potential utilization of acetate or fatty acids as a carbon source. In all MAGs, genes were found for the degradation of pyruvate (*aceEF*). Glycerol utilization (*ugpABE, malK*) was found for *Ca*. Id. esbjergensis and *Ca*. In. gracilis. Although *Ca*. Id. esbjergensis had genes for lactate (*lldDGEFP*) usage, none of the MAGs of *Ca*. Id. esbjergensis and *Ca*. In. gracilis had the complete pathway for mixed acid fermentation to lactate (*ppC, gltA, acnA, icd*). Only *Ca*. R. defluviihabitans encoded all genes for degradation of phosphoenolpyruvate to 2-oxoglutarate, which is part of mixed acid fermentation (47). Complete fatty acid catabolism was found in *Ca*. R. defluviihabitans (*fadAJD*, *aceA*, *acd*). All MAGs had, to some extent, the potential for fermentation of substrate, encoding the pyruvate:ferredoxin oxidoreductase (*porAB*), which works to convert pyruvate to acetyl-CoA under anoxic or microoxic conditions (48). Similarly, alanine dehydrogenase (*ald*) for the reduction of pyruvate to alanine was encoded.

Many bacteria in AS plants experience dynamic feast-famine conditions and have various storage compounds (49), such as glycogen and polyhydroxyalkanoates (PHA). These storage polymers are commonly associated with the phenotype of polyphosphate-accumulating organisms (PAOs). Under anoxic conditions, PAOs take up volatile fatty acids and store them in the form of PHA along with the degradation of poly-P and glycogen serving as energy sources. Under oxic conditions, PHA is oxidized to support cell growth and replenish poly-P and glycogen stores (50). All the MAGs belonging to *Ca*. R. defluviihabitans, *Ca*. Id. esbjergensis, and *Ca*. Intricatilinea gracilis encoded all genes required for PHA synthesis (*phaABC*). The full gene set for glycogen biosynthesis (*glgABC,* pathway M00854) was encoded, as well as several genes from the glycogen degradation pathway (M00855) and the alpha-amylase (AMY1A) gene, which may be used for glycogen/starch degradation to dextrin and maltose (51) (Fig. 7). Essential genes for poly-P accumulation (*pit*, *pstABCS*, *phoU*, *ppk*) were predicted in MAGs from *Ca*. R. defluviihabitans, *Ca*. Id. esbjergensis, and *Ca*. In. gracilis, suggesting potential PAO metabolism. However, these metabolic predictions could not be confirmed *in situ* by FISH–Raman microspectroscopy in AS samples from WWTPs with biological *P* removal (Fig. 6). None of the FISH-defined species showed any *in situ* intracellular storage of poly-P, PHA, or glycogen.

Most MAGs showed a potential for partial involvement in the denitrification process. The potential for nitrate and nitrite reduction was encoded in MAGs from *Ca*. R. defluviihabitans with the respiratory nitrate reductase (the *narGHJI* operon) and the two nitrite reductase genes *nrfAH* and *nirS* predicted, which shows its potential for ammonification. NrfAH is a periplasmic cytochrome *c* nitrite reductase used for respiratory ammonification (52), while *nirS* is a cytochrome *cd*_1_ nitrite reductase gene (53). They lacked genes for nitric oxide (*nor*) and nitrous oxide (*nos*) reductases. These results differed from other *Rubrivivax* species, such as *R. gelatinosus* encountered in AS systems for which genes for the reduction of NO_2_^–^ to N_2_ gas, but not NO_3_^–^ reduction genes, have been found (34). *Ca*. Id. esbjergensis encoded partial denitrification potential, with genes for nitrate reduction (*narGHI*), nitrite reduction (*nirS*), and nitric oxide (*norBC*) predicted, while no nitrous oxide (*nosZ*) reduction was encoded. The novel genus *Ca*. Intricatilinea presented only sporadically encoded denitrification genes including nitrate (*narG*) and nitrite (*nirK*) reductases.

The presence of photosynthesis gene clusters (PGC) was investigated, including bacteriochlorophyll biosynthesis genes (*bchCF*), carotenoid biosynthesis genes (*crtCDI*), and structural genes required for photosynthetic apparatuses (*pufABLM* and *puhA*). For *Ca*. Id. esbjergensis, a genomic potential for photosynthesis was predicted, but no presence of chlorophyll or carotenoids was observed *in situ* by FISH–Raman. *Ca*. R. defluviihabitans and *Ca*. Intricatilinea genera encoded the full PGC set of genes required for potential photosynthesis (Fig. S4), which was confirmed *in-situ* by FISH–Raman. A similar arrangement of photosynthetic genes is found in other members of the family *Burkholderiaceae*, including *Rubrivivax* (34) and *Rhodoferax* (54). It is unclear whether phototrophic capacity was inherited from an ancestral species, such as Alphaproteobacteria, or whether it was acquired by horizontal gene transfer (55). More research is needed to verify the photosynthetic metabolism of members of the family *Burkholderiaceae* and its potential role in AS systems.

**Fig 8:**
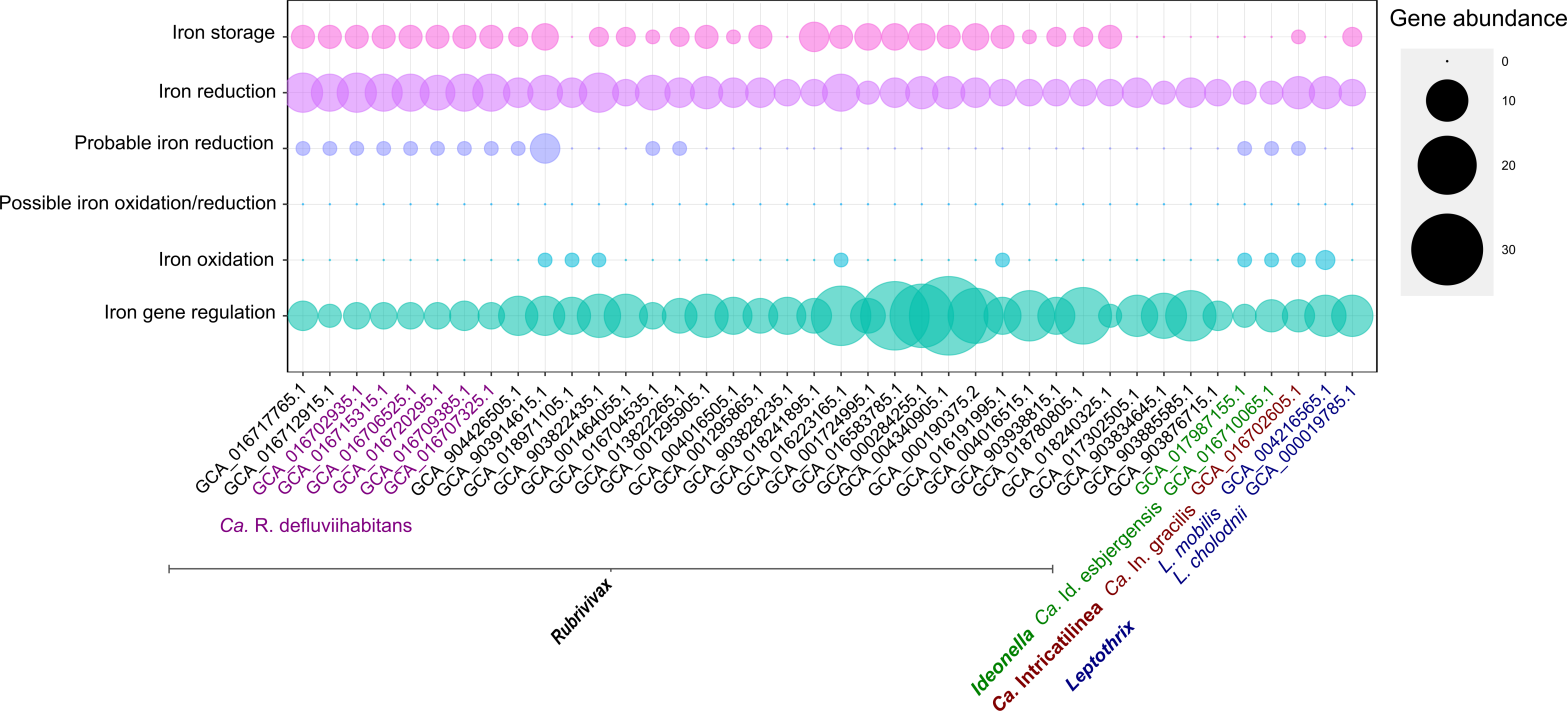
Genes involved in Fe transformations. The figure summarizes iron genes in *Ca*. R. defluviihabitans, *Ca*. Id. esbjergensis, and *Ca*. In. gracilis, all identified by FeGenie. No genes for magnetosome formation, siderophore transport, siderophore transport potential, and siderophore synthesis, iron acquisition heme oxygenase, iron acquisition heme transport, and iron acquisition/transport were detected in the MAGs and so are not shown.

The main iron cycling genes were identified by FeGenie (Fig. 8). All studied species exhibited potential for iron cycling. The genera *Ca*. Intricatilinea and *Ca*. Id. esbjergensis had a limited number of iron oxidation genes, whereas no iron oxidation genes were found in the six MAGs belonging to *Ca*. R. defluviihabitans. Iron reduction genes were identified across all MAGs under the category of “Iron reduction.” In addition, *Ca*. R. defluviihabitans and *Ca*. Intricatilinea also encoded genes involved in iron storage. Limited information exists regarding the physiological role of iron storage, including enhancing bacterial resistance to stress, such as low (56) iron concentrations. The observed potential for iron cycling capacity could be associated with the widespread use of ferric coagulants in AS systems (14, 57), but additional research is necessary to confirm this iron cycling *in situ* and to determine their contribution to the process in the AS ecosystems.

### Ecological importance of “*Leptothrix*” spp. in AS plants

Our study shows that members of the genus *Leptothrix* are not common in global AS plants in contrast to the general belief, partly due to classification errors because of the poor resolution of the 16S rRNA genes. Instead, the abundant species found in WWTPs belong to the genera *Ideonella*, *Rubrivivax*, and the novel genus *Ca*. Intricatilinea.

*Leptothrix* midas_s_884, defined by the 16S rRNA gene, was the most prevalent species found in European WWTPs and exhibited significant seasonal variation during the spring in Danish plants. However, its taxonomic classification could not be evaluated due to the lack of a MAG for this species. Unlike *Leptothrix* filaments, midas_s_884 cells were rod-shaped and widespread in the sludge flocs, thus not contributing to bulking as previously believed.

Members from the novel genus *Ca*. Intricatilinea exhibited a filamentous morphology that differs from the known sheathed filaments of *Leptothrix*, being short and thin. These filaments were located within the flocs, suggesting a structural role in floc formation. *Ca*. R. defluviihabitans and *Ca*. Id. esbjergensis displayed a rod-shaped morphology, as previously described for members of the *Rubrivivax* and *Ideonella* genera. All species had the potential for a wide range of carbon uptake, including sugars, fatty acids, and amino acids. They were found in nutrient removal plants, indicating a potential role in the removal of nitrogen and phosphorus. *Ca*. Id. esbjergensis was specifically abundant in nitrogen removal plants and exhibited potential for partial denitrification from nitrate to nitrous oxide. All species had the metabolic potential to be PAOs, as evidenced by the presence of genes encoding for the dynamic storage of PHA, glycogen, and poly-P (50). However, despite this potential, poly-P accumulation was not observed *in situ* by FISH–Raman. Why some species are seen to have the potential to be PAO but do not show the phenotype *in situ* is unknown (37), and further physiological studies are needed. Like other members of the *Burkholderiaceae* family, *Ca*. R. defluviihabitans and *Ca*. In. gracilis had genes for photosynthesis. Significant Raman spectra peaks associated with the presence of chlorophyll supported this, but further research is necessary to determine their role and extent of use in AS plants. All species demonstrated potential for iron cycling, as they encoded genes for iron reduction. Additionally, *Ca*. R. defluviihabitans and *Ca*. Intricatilinea had genes related to iron storage. Our study provides a deeper understanding of the most abundant “*Leptothrix*” spp. through genomic information and *in situ* validation. This approach can be applied to the study of other relevant microorganisms of uncertain taxonomy.

## MATERIALS AND METHODS

### Abundance and distribution

The abundance and distribution of “*Leptothrix*” spp. were studied in WWTPs using two 16S rRNA gene amplicon data sets: one including plants across the world from the MiDAS4 Global project (12) and one comprising long-term time-series from four Danish WWTPs (32). The data from both projects are available in the NCBI SRA under accession codes PRJNA728873 and PRJNA757616, respectively. More details about biomass sampling, DNA extraction, primers, and sequencing can be found in these papers. Time series analyses were carried out using weekly samples over 6 years from four Danish AS plants, as described by Peces et al. (32). Bioinformatic analysis was done in R v4.1.1 (58), R studio software v1.4.1717, (59) and visualized with ampvis2 v2.7.10 (60) and ggplot v3.2.1 (61) packages.

### FISH probe design and quantitative FISH

New FISH probes (Table 2) targeting the 16S rRNA of the *Leptothrix* spp., including midas_s_884, *Ca*. In.gracilis (midas_s_1298), and *Ca*. R. defluviihabitans (midas_s_81), were designed using ARB software v.6.0.6 (62) and the MiDAS4 database (12). Additionally, two new FISH probes targeting the 23S rRNA of species *Ca*. Id. esbjergensis (midas_s_130) were designed. 23S rRNA gene sequences were extracted from the HQ MAGs from Danish WWTP (26) using RNAmmer v1.2 (63) aligned with MAFFT (64); phylogenetic tree was calculated using IQ-TREE (65) using RAxML GTR algorithm with 100 bootstraps, and the probes were designed using ARB software. Coverage and specificity of the 23S rRNA FISH probes were evaluated *in silico* using testProbe (SILVA) (66). Specificity of all probes was validated *in silico* using MathFISH (67) to evaluate hybridization efficiencies of target and potentially weak non-target matches. Probe validation and optimization were performed on AS samples with high abundance of the target species, as evaluated from 16S rRNA amplicon data. Optimal formamide concentrations were determined by performing hybridization at the different formamide concentrations ranging from 0 to 70%, with an increment of 5%, where the average of relative fluorescence intensities of 50 cells was calculated using ImageJ (68). All probes were purchased from Biomers (Ulm, Germany) and labeled with cyanine-3 (Cy3), cyanine-5 (Cy5), or 6-FAM fluorochromes.

**TABLE 2:** FISH probes designed in this study^*d*^

Target group	Probe name	Sequence 5′−3′	Target site	*E. coli* position	Coverage MiDAS4.8	Non-target hits	FA%
***Leptothrix* midas_s_884**	**Lep884**	**5′-CCT GCT TTC ACC CTT AGG TC-3′**	**16S**	**183**	**22/38**	**9** ^*a*^	**45**
Comptetitor Lep884	Lep884_C1	5′-CCC GCT TTC ACC CTT AGG TC-3′	16S	184	NA^*e*^	NA	NA
Comptetitor Lep884	Lep884_C2	5′-CCT GCT TTC ACC CTM AGG TC-3′	16S	185	NA	NA	NA
Comptetitor Lep884	Lep884_C3	5′-CCT GCT TTC ACC CRT AGG TC-3′	16S	186	NA	NA	NA
***Ca.* Intricatilinea gracilis**	**Igra461**	**5′-CAT CAC CCC AGA GTA TTA GTC-3′**	**16S**	**461**	**11/11**	**2** ^*b*^	**30**
Competitor Igra461	Igra461_C1	5′-CAT KAC CCC AGA GTA TTA GTC-3′	16S	462	NA	NA	NA
Competitor Igra461	Igra461_C2	5′-CAT CAC CCC AGG GTA TTA GCC-3′	16S	463	NA	NA	NA
***Ca*. Rubrivivax defluviihabitans**	**Rdef820**	**5′-ACC TTT CCA ACA ACC AGT TGA CA-3′**	**16S**	**820**	**6/21**	**1** ^*c*^	**30**
Comptetitor Rdef820	Rdef820_C1	5′-ACC CTT CCA ACA ACC AGT TGA CA-3′	16S	820	NA	NA	NA
Comptetitor Rdef820	Rdef820_C2	5′-ACC TTC CCA ACA ACC AGT TGA CA-3′	16S	820	NA	NA	NA
Comptetitor Rdef820	Rdef820_C2	5′-ACC TTC CCA ACA ACC AGT TGA CA-3′	16S	820	NA	NA	NA
***Ca*. Ideonella esbjergensis**	**Iesb2460**	**5′-TCC ACA CTC GAC TTT TCT CAT A-3′**	**23S**	**2460**	**2/2**	**0**	**35**
	**Iesb1509**	**5′-GAG TAC AAG GCT GTT CCG ATT T-3′**	**23S**	**1509**	**2/2**	**0**	**35**

^*a*^ *Leptothrix* midas_s_345 (2), *Leptothrix* midas_s_39167 (1), *Leptothrix* midas_s_62607 (1), AAP99 midas_s_22449 (3), *Rhizobacter* midas_s_4800 (1), *Undibacterium* midas_s_40907 (1).

^*b*^ *Leptothrix* midas_s_9108 (2).

^*c*^ *Rubrivivax* midas_s_33271 (1).

^*d*^ Target species are in bold.

^*e*^ NA, not applicable.

FISH was performed as previously described (69) using the probes designed in this study. The quantitative FISH (qFISH) was performed for each species-specific probe (labeled with Cy3) and with EUBmix (labeled with Cy5) as universal probe. qFISH analysis was performed to estimate the biovolume fraction as percentage area of the total biovolume. In total, a set of 30 images was taken at 63× magnification using white light laser confocal microscope (Leica TCS SP8 X, Leica Microsystems, Germany) and analyzed with DAIME v2.2.2 software (70). Raman microspectroscopy was applied in combination with FISH, as previously described (36), to detect the intracellular storage polymers polyP, glycogen, PHA, and potential photosynthesis.

### Phylogenetic analyses

Phylogenetic analysis of the 16S rRNA gene sequences was performed using ARB software (62). A phylogenetic maximum likelihood tree was calculated using aligned 16S rRNA gene sequences retrieved from the MiDAS4 database (12) classified as *Leptothrix* and relevant HQ MAGs (26) using the GTR method and a 1,000× replicates bootstrap analysis.

For the phylogenomic analyses, genomes from “*Leptothrix*” spp., including midas_s_130, midas_s_81, and midas_s_1298, were identified in a set of HQ MAGS recovered from Danish WWTPs (26). Taxonomic classification at the phylogenetic level in relation to the genome taxonomy database and maximum likelihood placement was conducted using GTDB-Tk 2.1.0 (27) (Refseq release 207) “classify.” To determine the species [using a 95% average nucleotide identity (ANIb and ANIm) cutoff] and genus (using a 75–77% ANIm and ANIb cutoff), Pyani v0.2.11 (71) was used in combination with the GTDB classification. For the phylogenetic tree, multiple sequence alignments produced by GTDB-Tk 2.1.0 ‘de_novo_wf,’ of 120 concatenated single copy proteins trimmed to approx. 5,000 amino acids were used as input for IQ-TREE2 v2.0 (65) to create a maximum likelihood tree using WAG + G model and 1,000-replicate ultra-fast bootstrap analysis. Genomes representing *Leptothrix* and *Rubrivivax* were selected for inclusion in the bootstrapped tree based on the GTDB-Tk v2.1.0 tree and the paraphyletic clade incorporating all genomes of interest. The bootstrapped tree of *Leptothrix* and *Rubrivivax* was further examined and rooted in ARB v.6.0.6 (62), and iTOL v6 (71) was used for tree visualization with final aesthetic changes made in Inskape v1.2.2. Members of *Casimicrobium huifangae* (GCF_009746125.1), *Lautropia dentalis* (GCF_003892345.1) and *Pelomonas depolymerans* (GCA_003241055.1) were used as an outgroup.

### Metabolic reconstruction

The potential metabolism of the MAGs was annotated with KEGG orthology numbers (72) using EnrichM v0.5.0 (github.com/geronimp/enrichM) “annotate” using the EnrichM v10 database and the KO-annotated uniref100 database (73). Additionally, all MAGs were annotated using DRAM v1.3.5 (74) “annotate” with default settings to cross-validate EnrichM annotations. The presence of metabolic pathways was assumed to be present if the full set of genes in the KEGG module was encoded by both EnrichM and DRAM annotations. To identify the presence of the main iron metabolism genes, FeGenie with default settings was used (56).

### Protologues

Etymologies and protologues of the novel proposed species are given in Tables S1 to S3.

## SUPPLEMENTAL MATERIAL

Supplemental material aem.01485-24-s0001.pdf Figures S1 to S4; Tables S1 to S4.

Table S5 aem.01485-24-s0002.xlsx Gene names, associated KO numbers, and 16S rRNA gene copy numbers.

ASM does not own the copyrights to Supplemental Material that may be linked to, or accessed through, an article. The authors have granted ASM a non-exclusive, world-wide license to publish the Supplemental Material files. Please contact the corresponding author directly for reuse.

## References

[1] Fleming EJ, Langdon AE, Martinez-Garcia M, Stepanauskas R, Poulton NJ, Masland EDP, Emerson D. 2011. What’s new is old: resolving the identity of Leptothrix ochracea using single cell genomics, pyrosequencing and FISH. PLoS One 6:e17769. doi:10.1371/journal.pone.0017769 21437234 PMC3060100

[2] Spring S, Kampfer P, Ludwig W, Schleifer K-H. 1996. Polyphasic characterization of the genus Leptothrix: new descriptions of Leptothrix mobilis sp. nov. and Leptothrix discophora sp. nov. nom. rev. and emended description of Leptothrix cholodnii emend. Syst Appl Microbiol 19:634–643. doi:10.1016/S0723-2020(96)80036-1

[3] Spring S. 2006. The genera *Leptothrix* and *Sphaerotilus*, p 758–777. In The prokaryotes

[4] Eikelboom DH. 1975. Filamentous organisms observed in activated sludge. Water Res 9:365–388. doi:10.1016/0043-1354(75)90182-7

[5] van Veen WL, Mulder EG, Deinema MH. 1978. The Sphaerotilus-Leptothrix group of bacteria. Microbiol Rev 42:329–356. doi:10.1128/mr.42.2.329-356.1978 353479 PMC281433

[6] van Veen WL. 1973. Bacteriology of activated sludge, in particular the filamentous bacteria. Antonie Van Leeuwenhoek 39:189–205. doi:10.1007/BF02578852 4578055

[7] Meunier C, Henriet O, Schoonbroodt B, Boeur J-M, Mahillon J, Henry P. 2016. Influence of feeding pattern and hydraulic selection pressure to control filamentous bulking in biological treatment of dairy wastewaters. Bioresour Technol 221:300–309. doi:10.1016/j.biortech.2016.09.052 27643739

[8] Wagner M, Erhart R, Manz W, Amann R, Lemmer H, Wedi D, Schleifer KH. 1994. Development of an rRNA-targeted oligonucleotide probe specific for the genus Acinetobacter and its application for in situ monitoring in activated sludge. Appl Environ Microbiol 60:792–800. doi:10.1128/aem.60.3.792-800.1994 7512807 PMC201394

[9] van der Waarde J, Krooneman J, Geurkink B, van der Werf A, Eikelboom D, Beimfohr C, Snaidr J, Levantesi C, Tandoi V. 2002. Molecular monitoring of bulking sludge in industrial wastewater treatment plants. Water Sci Technol 46:551–558. 12216686

[10] Hirota K, Yokota Y, Sekimura T, Uchiumi H, Guo Y, Ohta H, Yumoto I. 2016. Bacterial communities in different locations, seasons and segments of a dairy wastewater treatment system consisting of six segments. J Environ Sci (China) 46:109–115. doi:10.1016/j.jes.2015.09.025 27521942

[11] Weissbrodt DG, Lochmatter S, Ebrahimi S, Rossi P, Maillard J, Holliger C. 2012. Bacterial selection during the formation of early-stage aerobic granules in wastewater treatment systems operated under wash-out dynamics. Front Microbiol 3:332. doi:10.3389/fmicb.2012.00332 22993513 PMC3440769

[12] Dueholm MS, Nierychlo M, Andersen KS, Rudkjøbing V, Knutsson S, Albertsen M, Nielsen PH, the MiDAS Global Consortium. 2021. MiDAS 4: a global catalogue of full-length 16S rRNA gene sequences and taxonomy for studies of bacterial communities in wastewater treatment plants. Nat. Commun 13:1908. doi:10.1101/2021.07.06.451231 PMC8989995 35393411

[13] Wágner DS, Peces M, Nierychlo M, Mielczarek AT, Thornberg D, Nielsen PH. 2022. Seasonal microbial community dynamics complicates the evaluation of filamentous bulking mitigation strategies in full-scale WRRFs. Water Res 216:118340. doi:10.1016/j.watres.2022.118340 35364352

[14] Seguel Suazo K, Dobbeleers T, Dries J. 2024. Bacterial community and filamentous population of industrial wastewater treatment plants in Belgium. Appl Microbiol Biotechnol 108:1–16. doi:10.1007/s00253-023-12822-8 38180550 PMC13496985

[15] Kunoh T, Yamamoto T, Sugimoto S, Ono E, Nomura N, Utada AS. 2021. Leptothrix cholodnii response to nutrient limitation. Front Microbiol 12:691563. doi:10.3389/fmicb.2021.691563 34248917 PMC8264430

[16] Mulder EG van Veen W 1963. Investigations on the Sphaerotilus -Lleptothrix group. Antonie Van Leeuwenhoek 29:121–153. doi:10.1007/BF02046045 14047145

[17] Dondero NC. 1975. The Sphaerotilus-Leptothrix group. Annu Rev Microbiol 29:407–428. doi:10.1146/annurev.mi.29.100175.002203 1180519

[18] Pringsheim G E. 1949. The filamentous bacteria Sphaerotilus, Leptothrix, Cladothrix, and their relation to iron and manganese. Phil Trans R Soc Lond B 233:453–482. doi:10.1098/rstb.1949.0002

[19] Schmidt B, Sánchez LA, Fretschner T, Kreps G, Ferrero MA, Siñeriz F, Szewzyk U. 2014. Isolation of Sphaerotilus-Leptothrix strains from iron bacteria communities in tierra del fuego wetlands. FEMS Microbiol Ecol 90:454–466. doi:10.1111/1574-6941.12406 25098830

[20] Fleming EJ, Woyke T, Donatello RA, Kuypers MMM, Sczyrba A, Littmann S, Emerson D. 2018. Insights into the fundamental physiology of the uncultured fe-oxidizing bacterium leptothrix ochracea. Appl Environ Microbiol 84:e02239-17. doi:10.1128/AEM.02239-17 29453262 PMC5930342

[21] Emerson D, Fleming EJ, McBeth JM. 2010. Iron-oxidizing bacteria: an environmental and genomic perspective. Annu Rev Microbiol 64:561–583. doi:10.1146/annurev.micro.112408.134208 20565252

[22] Lechner U, Brodkorb D, Geyer R, Hause G, Härtig C, Auling G, Fayolle-Guichard F, Piveteau P, Müller RH, Rohwerder T. 2007. Aquincola tertiaricarbonis gen. nov., sp. nov., a tertiary butyl moiety-degrading bacterium. Int J Syst Evol Microbiol 57:1295–1303. doi:10.1099/ijs.0.64663-0 17551046

[23] Manaia CM, Nunes OC, Nogales B. 2003. Caenibacterium thermophilum gen. nov., sp. nov., isolated from a thermophilic aerobic digester of municipal sludge. Int J Syst Evol Microbiol 53:1375–1382. doi:10.1099/ijs.0.02622-0 13130021

[24] Siering PL, Ghiorse WC. 1996. Phylogeny of the Sphaerotilus-Leptothrix group inferred from morphological comparisons, genomic fingerprinting, and 16S ribosomal DNA sequence analyses. Int J Syst Bacteriol 46:173–182. doi:10.1099/00207713-46-1-173 8573492

[25] Liu Y, Du J, Pei T, Du H, Feng G-D, Zhu H. 2022. Genome-based taxonomic classification of the closest-to-Comamonadaceae group supports a new family Sphaerotilaceae fam. nov. and taxonomic revisions. Syst Appl Microbiol 45:126352. doi:10.1016/j.syapm.2022.126352 36063784

[26] Singleton CM, Petriglieri F, Kristensen JM, Kirkegaard RH, Michaelsen TY, Andersen MH, Kondrotaite Z, Karst SM, Dueholm MS, Nielsen PH, Albertsen M. 2021. Connecting structure to function with the recovery of over 1000 high-quality metagenome-assembled genomes from activated sludge using long-read sequencing. Nat Commun 12:2009. doi:10.1038/s41467-021-22203-2 33790294 PMC8012365

[27] Chaumeil PA, Mussig AJ, Hugenholtz P, Parks DH. 2020. GTDB-Tk: a toolkit to classify genomes with the genome taxonomy database. Bioinformatics 36:1925–1927. doi:10.1093/bioinformatics/btz848 PMC7703759 31730192

[28] Imhoff JF. 2015. Incertae sedis V. *Rubrivivax*, p 1–8p. In Bergey’s Manual of Systematics of Archaea and Bacteria

[29] Malmqvist Å, Moore ERB, Ternström A. 2015. Incertae sedis II. *Ideonella*, p 1–4. In Bergey’s Manual of Systematics of Archaea and Bacteria

[30] Barco RA, Garrity GM, Scott JJ, Amend JP, Nealson KH, Emerson D. 2020. A genus definition for bacteria and archaea based on A standard genome relatedness index. MBio 11:e02475–19. 31937639 10.1128/mBio.02475-19 PMC6960282

[31] Parks DH, Chuvochina M, Chaumeil P-A, Rinke C, Mussig AJ, Hugenholtz P. 2020. A complete domain-to-species taxonomy for bacteria and archaea. Nat Biotechnol 38:1079–1086. doi:10.1038/s41587-020-0501-8 32341564

[32] Peces M, Dottorini G, Nierychlo M, Andersen KS, Dueholm MKD, Nielsen PH. 2022. Microbial communities across activated sludge plants show recurring species-level seasonal patterns. ISME Commun 2:18. doi:10.1038/s43705-022-00098-4 37938743 PMC9723569

[33] Li H, Yang Q, Li J, Gao H, Li P, Zhou H. 2015. The impact of temperature on microbial diversity and AOA activity in the Tengchong Geothermal Field, China. Sci Rep 5:17056. doi:10.1038/srep17056 26608685 PMC4660298

[34] Nagashima S, Kamimura A, Shimizu T, Nakamura-Isaki S, Aono E, Sakamoto K, Ichikawa N, Nakazawa H, Sekine M, Yamazaki S, Fujita N, Shimada K, Hanada S, Nagashima KVP. 2012. Complete genome sequence of phototrophic betaproteobacterium Rubrivivax gelatinosus IL144. J Bacteriol 194:3541–3542. doi:10.1128/JB.00511-12 22689232 PMC3434721

[35] Chen WM, Chen LC, Sheu DS, Tsai JM, Sheu SY. 2020. Ideonella livida sp. nov., isolated from a freshwater lake. Int J Syst Evol Microbiol 70:4942–4950. doi:10.1099/ijsem.0.004363 32749952

[36] Fernando EY, McIlroy SJ, Nierychlo M, Herbst F-A, Petriglieri F, Schmid MC, Wagner M, Nielsen JL, Nielsen PH. 2019. Resolving the individual contribution of key microbial populations to enhanced biological phosphorus removal with Raman-FISH. ISME J 13:1933–1946. doi:10.1038/s41396-019-0399-7 30894691 PMC6776032

[37] Petriglieri F, Singleton C, Peces M, Petersen JF, Nierychlo M, Nielsen PH. 2021. “Candidatus Dechloromonas phosphoritropha” and “Candidatus Dechloromonas phosphorivorans”, novel polyphosphate accumulating organisms abundant in wastewater treatment systems. ISME J 15:3605–3614. doi:10.1038/s41396-021-01029-2 34155336 PMC8630035

[38] Heraud P, Beardall J, McNaughton D, Wood BR. 2007. In vivo prediction of the nutrient status of individual microalgal cells using Raman microspectroscopy. FEMS Microbiol Lett 275:24–30. doi:10.1111/j.1574-6968.2007.00861.x 17854469

[39] Jehlička J, Edwards HGM, Oren A. 2014. Raman spectroscopy of microbial pigments. Appl Environ Microbiol 80:3286–3295. doi:10.1128/AEM.00699-14 24682303 PMC4018853

[40] Maquelin K, Hoogenboezem T, Jachtenberg JW, Dumke R, Jacobs E, Puppels GJ, Hartwig NG, Vink C. 2009. Raman spectroscopic typing reveals the presence of carotenoids in Mycoplasma pneumoniae. Microbiology (Reading) 155:2068–2077. doi:10.1099/mic.0.026724-0 19383695

[41] Collins AM, Xin Y, Blankenship RE. 2009. Pigment organization in the photosynthetic apparatus of Roseiflexus castenholzii. Biochimica et Biophysica Acta (BBA) - Bioenergetics 1787:1050–1056. doi:10.1016/j.bbabio.2009.02.027 19272352

[42] Agalidis I, Mattioli T, Reiss-Husson F. 1999. Spirilloxanthin is released by detergent from Rubrivivax gelatinosus reaction center as an aggregate with unusual spectral properties. Photosyn Res 62:31–42. doi:10.1023/A:1006384113191

[43] Vítek P, Cámara-Gallego B, Edwards HGM, Jehlička J, Ascaso C, Wierzchos J. 2013. Phototrophic community in gypsum crust from the Atacama desert studied by raman spectroscopy and microscopic imaging. Geomicrobiol J 30:399–410. doi:10.1080/01490451.2012.697976

[44] Bjerg JT, Boschker HTS, Larsen S, Berry D, Schmid M, Millo D, Tataru P, Meysman FJR, Wagner M, Nielsen LP, Schramm A. 2018. Long-distance electron transport in individual, living cable bacteria. Proc Natl Acad Sci USA 115:5786–5791. doi:10.1073/pnas.1800367115 29735671 PMC5984516

[45] Berg JS, Schwedt A, Kreutzmann A-C, Kuypers MMM, Milucka J. 2014. Polysulfides as intermediates in the oxidation of sulfide to sulfate by Beggiatoa spp. Appl Environ Microbiol 80:629–636. doi:10.1128/AEM.02852-13 24212585 PMC3911116

[46] El Gheriany IA, Bocioaga D, Hay AG, Ghiorse WC, Shuler ML, Lion LW. 2009. Iron requirement for Mn(II) oxidation by Leptothrix discophora SS-1. Appl Environ Microbiol 75:1229–1235. doi:10.1128/AEM.02291-08 19114505 PMC2648177

[47] Friesen JD. 1988. Escherichia coli and Salmonella typhimurium: cellular and molecular biology. Science 240:1678–1681. 17745224 10.1126/science.240.4859.1678

[48] Ragsdale SW. 2003. Pyruvate ferredoxin oxidoreductase and its radical intermediate. Chem Rev 103:2333–2346. doi:10.1021/cr020423e 12797832

[49] van Loosdrecht MCM, Pot MA, Heijnen JJ. 1997. Importance of bacterial storage polymers in bioprocesses. Water Sci Technol 35:41–47. doi:10.2166/wst.1997.0008

[50] Kristiansen R, Nguyen HTT, Saunders AM, Nielsen JL, Wimmer R, Le VQ, McIlroy SJ, Petrovski S, Seviour RJ, Calteau A, Nielsen KL, Nielsen PH. 2013. A metabolic model for members of the genus Tetrasphaera involved in enhanced biological phosphorus removal. ISME J 7:543–554. doi:10.1038/ismej.2012.136 23178666 PMC3578573

[51] Janecek S. 1997. Alpha-amylase family: molecular biology and evolution. Prog Biophys Mol Biol 67:67–97. doi:10.1016/s0079-6107(97)00015-1 9401418

[52] Simon J. 2002. Enzymology and bioenergetics of respiratory nitrite ammonification. FEMS Microbiol Rev 26:285–309. doi:10.1111/j.1574-6976.2002.tb00616.x 12165429

[53] Härtig E, Zumft WG. 1999. Kinetics of nirS expression (cytochrome cd1 nitrite reductase) in Pseudomonas stutzeri during the transition from aerobic respiration to denitrification: evidence for a denitrification-specific nitrate- and nitrite-responsive regulatory system. J Bacteriol 181:161–166. doi:10.1128/JB.181.1.161-166.1999 9864326 PMC103545

[54] Imhoff JF, Rahn T, Künzel S, Neulinger SC. 2017. Photosynthesis is widely distributed among proteobacteria as demonstrated by the phylogeny of PufLM reaction center proteins. Front Microbiol 8:2679. doi:10.3389/fmicb.2017.02679 29472894 PMC5810265

[55] Cardona T. 2019. Thinking twice about the evolution of photosynthesis. Open Biol 9:180246. doi:10.1098/rsob.180246 30890026 PMC6451369

[56] Garber AI, Nealson KH, Okamoto A, McAllister SM, Chan CS, Barco RA, Merino N. 2020. FeGenie: a comprehensive tool for the identification of iron genes and iron gene neighborhoods in genome and metagenome assemblies. Front Microbiol 11. doi:10.3389/fmicb.2020.00037 PMC7005843 32082281

[57] Rasmussen H, Nielsen PH. 1996. Iron reduction in activated sludge measured with different extraction techniques. Water Res 30:551–558. doi:10.1016/0043-1354(95)00203-0

[58] Core TR. 2020. R: A language and environment for statistical computing. R Foundation for Statistical Computing, Vienna, Austria.

[59] Team R. 2015. RStudio: integrated development for R. Boston, MA

[60] Andersen KS, Kirkegaard RH, Karst SM, Albertsen M. 2018. Ampvis2: an R package to analyse and visualise 16S rRNA amplicon data. Bioinformatics. doi:10.1101/299537

[61] Wickham H. 2009. Ggplot2: elegant graphics for data analysis. Springer Science & Business Media, New York, NY.

[62] Ludwig W, Strunk O, Westram R, Richter L, Meier H, Buchner A, Lai T, Steppi S, Jobb G, et al.. 2004. ARB: a software environment for sequence data. Nucleic Acids Res 32:1363–1371. doi:10.1093/nar/gkh293 14985472 PMC390282

[63] Lagesen K, Hallin P, Rødland EA, Staerfeldt H-H, Rognes T, Ussery DW. 2007. RNAmmer: consistent and rapid annotation of ribosomal RNA genes. Nucleic Acids Res 35:3100–3108. doi:10.1093/nar/gkm160 17452365 PMC1888812

[64] Katoh K, Standley DM. 2013. MAFFT multiple sequence alignment software version 7: improvements in performance and usability. Mol Biol Evol 30:772–780. doi:10.1093/molbev/mst010 23329690 PMC3603318

[65] Nguyen L-T, Schmidt HA, von Haeseler A, Minh BQ. 2015. IQ-TREE: a fast and effective stochastic algorithm for estimating maximum-likelihood phylogenies. Mol Biol Evol 32:268–274. doi:10.1093/molbev/msu300 25371430 PMC4271533

[66] Quast C, Pruesse E, Yilmaz P, Gerken J, Schweer T, Yarza P, Peplies J, Glöckner FO. 2013. The SILVA ribosomal RNA gene database project: improved data processing and web-based tools. Nucleic Acids Res 41:D590–6. doi:10.1093/nar/gks1219 23193283 PMC3531112

[67] Yilmaz LS, Parnerkar S, Noguera DR. 2011. mathFISH, a web tool that uses thermodynamics-based mathematical models for in silico evaluation of oligonucleotide probes for fluorescence in situ hybridization. Appl Environ Microbiol 77:1118–1122. doi:10.1128/AEM.01733-10 21148691 PMC3028703

[68] Schneider CA, Rasband WS, Eliceiri KW. 2012. NIH image to ImageJ: 25 years of image analysis. Nat Methods 9:671–675. doi:10.1038/nmeth.2089 22930834 PMC5554542

[69] Daims H, Stoecker K, Wagner M. 2005. Fluorescence in situ hybridization for the detection of prokaryotes, p 213–239. In Osborn AM, Smith CJ (ed), Molecular microbial ecology. Taylor & Francis, New York.

[70] Daims H, Lücker S, Wagner M. 2006. Daime, a novel image analysis program for microbial ecology and biofilm research. Environ Microbiol 8:200–213. doi:10.1111/j.1462-2920.2005.00880.x 16423009

[71] Letunic I, Bork P. 2021. Interactive Tree Of Life (iTOL) v5: an online tool for phylogenetic tree display and annotation. Nucleic Acids Res 49:W293–W296. doi:10.1093/nar/gkab301 33885785 PMC8265157

[72] Kanehisa M, Goto S. 2000. KEGG: kyoto encyclopedia of genes and genomes. Nucleic Acids Res 28:27–30. doi:10.1093/nar/28.1.27 10592173 PMC102409

[73] Suzek BE, Wang Y, Huang H, McGarvey PB, Wu CH, Consortium U. 2015. UniRef clusters: a comprehensive and scalable alternative for improving sequence similarity searches. Bioinformatics 31:926–932. doi:10.1093/bioinformatics/btu739 25398609 PMC4375400

[74] Shaffer M, Borton MA, McGivern BB, Zayed AA, La Rosa SL, Solden LM, Liu P, Narrowe AB, Rodríguez-Ramos J, Bolduc B, Gazitúa MC, Daly RA, Smith GJ, Vik DR, Pope PB, Sullivan MB, Roux S, Wrighton KC. 2020. DRAM for distilling microbial metabolism to automate the curation of microbiome function. Nucleic Acids Res 48:8883–8900. doi:10.1093/nar/gkaa621 32766782 PMC7498326

