# Supplementary material for: Diversity and abundance of filamentous and non-filamentous “Leptothrix” in global wastewater treatment plants
Source: Appl Environ Microbiol. 2025 Feb 14;91(3):e01485-24. doi: 10.1128/aem.01485-24 (PMC11921362; doi:10.1128/aem.01485-24)
Supplement: Supplemental material — Figures S1 to S4; Tables S1 to S4. [file aem.01485-24-s0001.pdf]

Supplemental Information

Diversity and abundance of filamentous and non-filamentous "*Leptothrix*" in global wastewater treatment plants

Karina Seguel Suazo [1], Marta Nierychlo [2], Zivile Kondrotaite [2], Francesca Petriglieri [2], Miriam Peces [2], Caitlin Singleton [2], Jan Dries [1], Per H. Nielsen [2]

[1] Biochemical Wastewater Valorization and Engineering (BioWAVE), Faculty of Applied Engineering, University of Antwerp, Antwerp, Belgium.

[2] Center for Microbial Communities, Department of Chemistry and Bioscience, Aalborg University, Aalborg, Denmark.

Supplementary Figures

- **FIG S1.** ANIb of MAGs.
- **FIG S2.** Heatmap comparing the relative abundance of most abundant species within "*Leptothrix*" spp., based on different primers.
- **FIG S3.** Average relative abundance of most abundant "*Leptothrix*" spp. in AS WWTP in Denmark.
- **FIG S4.** Functional potential of *Leptothrix*, *Ca*. Intricatilinea, *Ideonella*, and *Rubrivivax.*

Supplementary Tables

- **Table S1.** Protologues for *Candidatus* Rubrivivax defluviihabitans
- **Table S2**. Protologues for *Candidatus* Ideonella esbjergensis
- **Table S3.** Protologues for *Candidatus* Intricatilinea gracilis
- **Table S4.** Exact p-values for Kruskal-Wallis statistical test comparing process type, industrial load, and temperature range.
- **Table S5:** List of gene names, associated KO numbers and 16S rRNA copy numbers. See separate excel file (SupplementaryTable_S5)

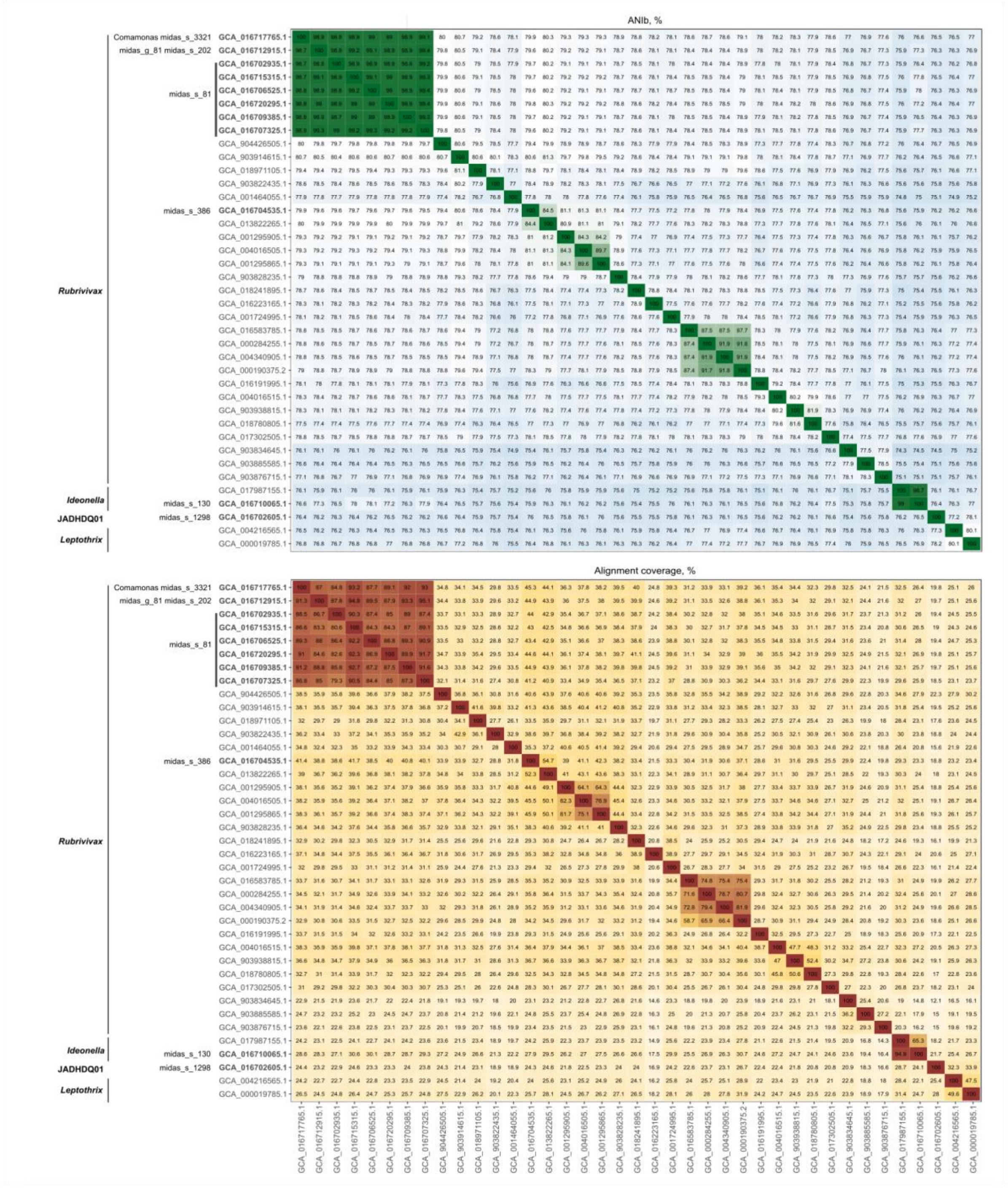

FIG S1. ANIb of MAGs. A**)** ANIb of the MAGs belonging to *Ca*. Rubrivivax defluviihabitans (midas_s_81), *Ca*. Ideonella esbjergensis (midas_s_130), *Ca*. Intricatilinea (midas_s_1298), and *Leptothrix* isolates/genomes; B) Alignment coverage of ANIb. Representative MAGs of each species are in bold.

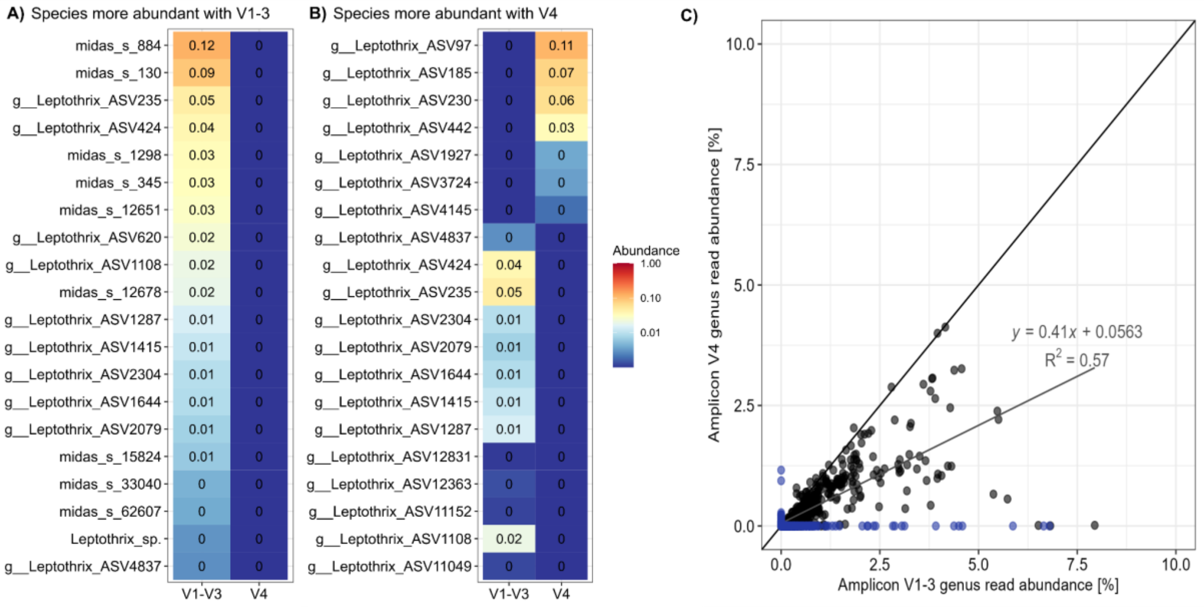

FIG S2. Heatmap comparing the relative abundance of most abundant species within "*Leptothrix*" spp., based on different primers. A) V1-V3 and B) V4 region of 16S rRNA gene amplicon data (species taxonomy is shown if available, with very few identified by V4), and C) scatter plot comparing the relative abundance based on V1-V3 and V4 region of 16S rRNA gene amplicon data (samples not present in one of the datasets are shown in blue). Data comes from the MiDAS Global project including 483 AS WWTPs from around the world (Dueholm et al., 2022).

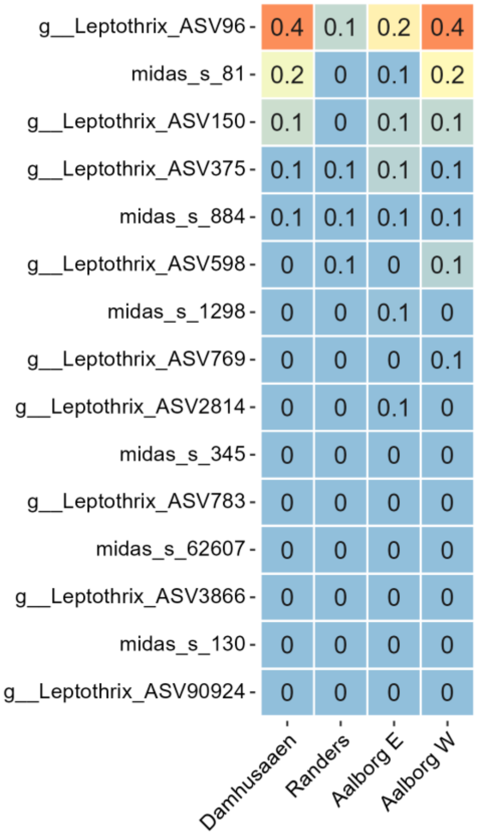

FIG S3. Average relative abundance of most abundant "*Leptothrix*" spp. in 4 AS WWTP in Denmark. Values represent averages from min. 161 samples (Damhusaaen) to max. 271 samples (Aalborg W) taken at regular intervals in the period from 2015 to 2020.

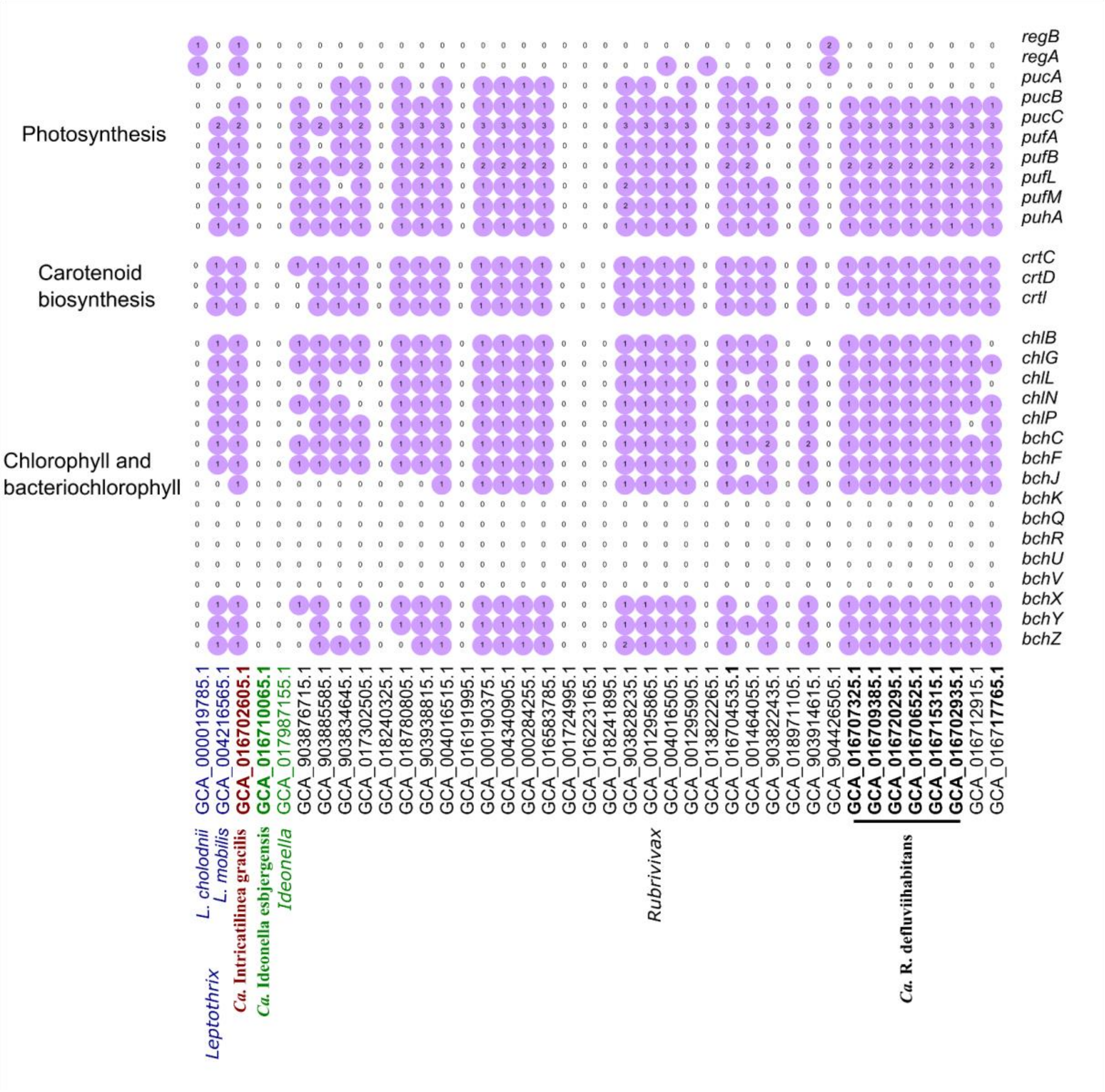

FIG S4. Functional potential of *Leptothrix*, *Ca*. Intricatilinea, *Ideonella*, and *Rubrivivax*. Focus on structural genes required for photosynthetic apparatuses, carotenoid biosynthesis, and bacteriochlorophyll biosynthesis genes. The studied species of *Ca*. Intricatilinea gracilis, *Ca*. Ideonella esbjergensis, and *Ca*. R. defluviihabitans are in bold.

Supplementary Tables

Taxonomic proposal and protologue tables

Table S1. Protologues for *Candidatus* Rubrivivax defluviihabitans

Species name	*Candidatus* Rubrivivax defluviihabitans
Genus name	*Rubrivivax*
Specific epithet	defluviihabitans
Type species of the genus	*Rubrivivax albus*<br>*Rubrivivax benzoatilyticus*<br>*Rubrivivax gelatinosus*<br>*Rubrivivax indolicus*
Genus status	Validly published (Taxonomy ID: 28067)
Species etymology	"*Candidatus* Rubrivivax defluviihabitans" (de.flu.vi.i.ha'bi.tans. L. neut. n. *defluvium*, sewage; L. pres. part. *habitans*, inhabitant; N.L. part. adj. *defluviihabitans,* inhabiting sewage).
Species status	sp. nov.
Designation of the type MAG	GCA_016709385.1
MAG/SAG accession number	GCA_016709385.1
Genome status	High-quality draft
Genome size	4,688,135
GC mol %	69.44
Country of origin	Denmark
Region of origin	Egaa
Source of sample	Activated sludge
Geographical location	Egaa
Latitude	56.21314 N
Longitude	10.242467 E
Depth	N/A
Altitude	N/A
Temperature of the sample	Mesophilic
pH of the sample	N/A
Relationship to oxygen	Facultative anaerobe
Energy metabolism	Potentially utilizing a range of substrates including amino acids and fatty acids. Capable of polyphosphate accumulation cycling, with the potential for photosynthetic metabolism.
Assembly	1 sample
Sequencing technology	Oxford Nanopore PromethION
Binning software used	MaxBin2
Assembly software used	CANU v.1.8
Habitat	Full-scale nutrient removal wastewater treatment plant
Miscellaneous, extraordinary features relevant for the description	Rod-shaped cells, with mostly short rods (0.4–0.6 µm x 0.8–1.3 µm), radially symmetric with rounded ends observed mostly scattered within the flocs and in some cases, they were observed to cluster in microcolonies

Table S2. **Protologues for *Candidatus* Ideonella esbjergensis**

Species name	*Candidatus* Ideonella esbjergensis
Genus name	Ideonella
Specific epithet	esbjergensis
Type species of the genus	*Ideonella alba*<br>*Ideonella aquatica*<br>*Ideonella azotifigens*<br>*Ideonella benzenivorans*<br>*Ideonella dechloratans*<br>*Ideonella livida*<br>*Ideonella paludis*<br>*Ideonella sakaiensis*
Genus status	Validly published (Taxonomy ID: 36862)
Species etymology	"*Candidatus* Ideonella esbjergensis" (es.bjerg.en´sis. N.L. fem. adj. *esbjergensis*, pertaining to Esbjerg)
Species status	sp. nov.
Designation of the type MAG	GCA_016710065.1
MAG/SAG accession number	GCA_016710065.1
Genome status	High-quality draft
Genome size	5,531,576
GC mol %	66.32
Country of origin	Denmark
Region of origin	Esbjerg
Source of sample	Activated sludge
Geographical location	Esbjerg W
Latitude	55.488097
Longitude	8.430505
Depth	N/A
Altitude	N/A
Temperature of the sample	Mesophilic
pH of the sample	N/A
Relationship to oxygen	Facultative anaerobe
Energy metabolism	Potentially utilizing a range of substrates including some sugars, amino acids and fatty acids. Likely capable of polyphosphate accumulation.
Assembly	1 sample
Sequencing technology	Oxford Nanopore PromethION
Binning software used	MetaBAT2
Assembly software used	CANU v.1.8
Habitat	Full-scale nutrient removal wastewater treatment plant
Miscellaneous, extraordinary features relevant for the description	Straight and long rod shaped cells (0.2–0.6 µm x 1.3–3.2 µm), mostly scattered within the sludge flocs.

Table S3. Protologues for *Candidatus* Intricatilinea gracilis

Species name	*Candidatus* Intricatilinea gracilis
Genus name	*Candidatus* Intricatilinea
Specific epithet	gracilis
Type species of the genus	*Candidatus* Intricatilinea gracilis
Genus status	*Candidatus*
Species etymology	"*Candidatus* Intricatilinea (In.tri.ca.ti.li´ne.a. L. past part. *intricatus,* tangled; L. fem. n. *linea*, line; N.L. fem. n. *Intricatilinea*, an entangled filamentous organism).
Species status	sp. nov.
Designation of the type MAG	GCA_016710065.1
MAG/SAG accession number	GCA_016710065.1
Genome status	High-quality draft
Genome size	5,531,576 bp
GC mol %	66.32%
Country of origin	Denmark
Region of origin	Esbjerg
Source of sample	Activated sludge
Geographical location	Esbjerg W
Latitude	55.488097
Longitude	8.430505
Depth	N/A
Altitude	N/A
Temperature of the sample	Mesophilic
pH of the sample	N/A
Relationship to oxygen	Facultative anaerobe
Energy metabolism	Potentially utilizing a range of substrates including some sugars, amino acids and fatty acids. Capable of polyphosphate accumulation, with the potential for photosynthetic metabolism.
Assembly	1 sample
Sequencing technology	Oxford Nanopore PromethION
Binning software used	MetaBAT2
Assembly software used	CANU v.1.8
Habitat	Full-scale nutrient removal wastewater treatment plant
Miscellaneous, extraordinary features relevant for the description	Thin filaments (0.4-0.6 µm x 15–57 µm), that were mostly located within the flocs.

Table S4. Exact p-values for Kruskal-Wallis statistical test comparing process type, industrial load, and temperature range.

	Process type	Industrial load	Temperature range
Ca. Intricatilinea gracilis	0.000000008653	0.0000000744	< 2.2e-16
Ca. Ideonella esbjergensis	0.000000001	0.001108	< 2.2e-16
Ca. Rubrivivax defluviihabitans	0.0000002831	0.6368	0.06386
Leptothrix midas_s_884	< 2.2e-16	< 2.2e-16	< 2.2e-16

Table S5: List of gene names,associated KO numbers and 16S rRNA copy numbers

See separate excel file (SupplementaryTable_S5)
